# Practical Synthesis of 7-Bromo-4-chloro-1*H*-indazol-3-amine: An Important Intermediate to Lenacapavir

**DOI:** 10.3390/molecules29122705

**Published:** 2024-06-07

**Authors:** Naeem Asad, Michael Lyons, Shirley Muniz Machado Rodrigues, Justina M. Burns, Thomas D. Roper, G. Michael Laidlaw, Saeed Ahmad, B. Frank Gupton, Douglas Klumpp, Limei Jin

**Affiliations:** Medicines for All Institute, Virginia Commonwealth University, Richmond, VA 23284-3068, USA; asadn@vcu.edu (N.A.); lyonsmr2@vcu.edu (M.L.); munizmachas@vcu.edu (S.M.M.R.); burnsj4@vcu.edu (J.M.B.); tdroper@vcu.edu (T.D.R.); glaidlaw@vcu.edu (G.M.L.); sahmad@vcu.edu (S.A.); bfgupton@vcu.edu (B.F.G.); klumppd@vcu.edu (D.K.)

**Keywords:** Lenacapavir, indazol-3-amine, hydrazine, regioselective cyclization, process development

## Abstract

7-Bromo-4-chloro-1*H*-indazol-3-amine is a heterocyclic fragment used in the synthesis of Lenacapavir, a potent capsid inhibitor for the treatment of HIV-1 infections. In this manuscript, we describe a new approach to synthesizing 7-bromo-4-chloro-1*H*-indazol-3-amine from inexpensive 2,6-dichlorobenzonitrile. This synthetic method utilizes a two-step sequence including regioselective bromination and heterocycle formation with hydrazine to give the desired product in an overall isolated yield of 38–45%. The new protocol has been successfully demonstrated on hundred-gram scales without the need for column chromatography purification. This new synthesis provides a potential economical route to the large-scale production of this heterocyclic fragment of Lenacapavir.

## 1. Introduction

**Figure 1:**
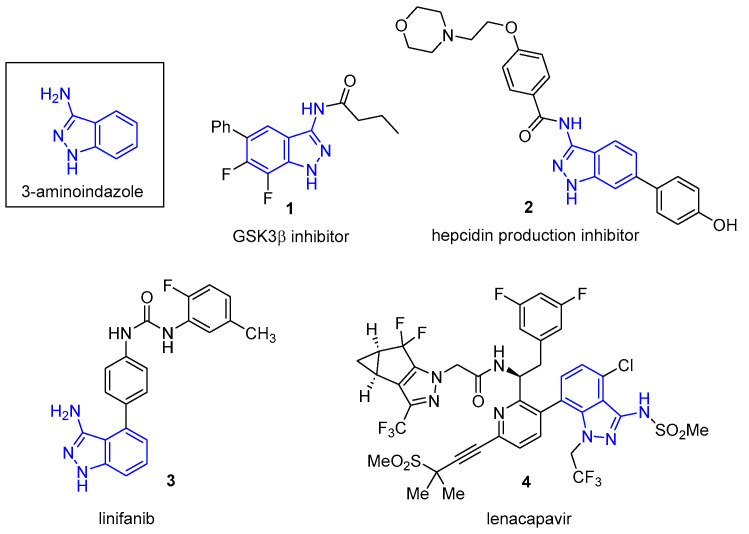
Biologically active 3-aminoindazoles.

3-Aminoindazoles are a privileged class of heterocyclic structures common to many biologically active compounds. For example, this structure is found in the glycogen synthase 3β inhibitor **1**, a substance having potential for the treatment of Alzheimer’s disease (Figure 1) [1]. The 3-aminoindazole (**2**) was developed as a possible treatment for iron deficiency [2], while linifanib (**3**) is a potent tyrosine kinase receptor inhibitor used to suppress tumor growth [3]. Recently, Gilead developed Lenacapavir (**4**), which exhibits high potency in the treatment of human immunodeficiency virus (HIV) [4,5]. Among the synthetic routes used to prepare 3-aminoindazoles, S_N_Ar chemistry has often been utilized. For example, in the synthesis of Lenacapavir, 3-bromo-6-chloro-2-fluorobenzonitrile (**5**) reacts with hydrazine to provide the 3-aminoindazole (**6**) in a 90% yield (Equation (1)) [4].

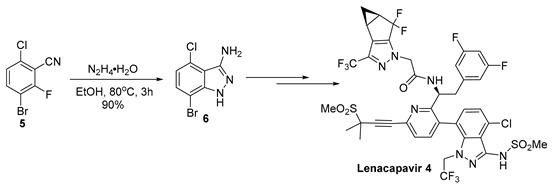
(1)

A similar transformation is achieved with 2,6-dichlorobenzonitrile (**7**) and hydrazine to form the 3-aminoindazole leading to linifanib (**3**) [6]. While 2-chloro and 2-fluorobenzonitriles are commonly used in this method, 2-bromobenzonitriles have provided 3-aminoindazoles through transition metal-catalyzed reactions [7]. The 3-aminoindazole scaffold has also been prepared from S_N_Ar chemistry with hydrazine and a 2-nitrobenzonitrile [8]. Besides these approaches, synthetic routes to 3-aminoindazoles have utilized 2-fluorocarboxylic acids (via thioamides), ortho-haloaryl hydrazines in Pd-catalyzed cyclization, the C-N coupling of halogenated indazoles, intramolecular N-N coupling reactions, and others [9,10,11].

With the promising results from the clinical trials of Lenacapavir, it is vitally important that this substance can be prepared economically on a large scale [12]. While the 3-aminoindazole fragment of Lenacapavir has been prepared on a pilot plant scale (Equation (1)), the synthetic route utilizes a costly 3-bromo-6-chloro-2-fluorobenzonitrile (**5**) [13] and the heterocycle synthesis leads to the elimination of HF [6]. We envisioned that 3-bromo-2,6-dichlorobenzonitrile (**8**) might be used as precursor to synthesize 3-aminoindazole (**6**) using a similar S_N_Ar cyclization with hydrazine, perhaps allowing for the use of the less expensive 2,6-dichlorobenzonitrile as the starting material. In the present manuscript, we describe our work in the preparation of the 3-aminoindazole fragment of Lenacapavir. The new chemistry leverages a highly regioselective bromination and selective cyclization step to provide an efficient route to the functionalized heterocycle. 

## 2. Results

**Scheme 1:**
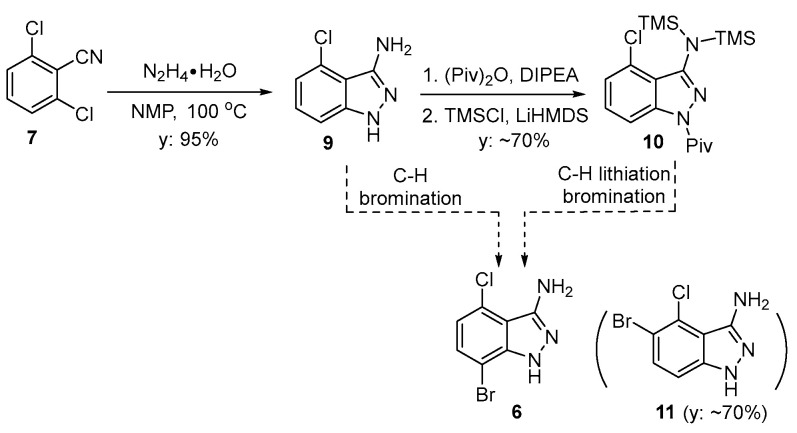
Initial attempt at synthesis of **6**.

Our initial efforts to produce 7-bromo-4-chloro-1*H*-indazol-3-amine (**6**) involved forming the 3-aminoindazole ring first and then brominating the heterocyclic product. In accordance with the literature, the 3-aminoindazole (**9**) was prepared from the condensation of **7** with hydrazine hydrate in a 95% yield (Scheme 1) [6]. The direct bromination of compound **9** was not successful. The treatment of **9** with NBS afforded the undesired regioisomer **11** as the major product (based on ^1^HNMR, GCMS).

As another approach to functionalize the 7-position, we sought to carry out the regioselective functionalization of protected 3-aminoindazole derivative **10**. Treating **10** with different organolithium reagents, such as n-butyllithium (BuLi), lithium diisopropylamide (LDA), or lithium bis(trimethylsilyl)amide (LiHMDS), followed by treatment with bromine, resulted in a complex mixture of products. The functionalization of the 7-position was also attempted with the borylation of the lithiated products of **10**, using B(OMe)_3_ and B(OiPr)_3_ after a reaction with the organolithium base. This approach also failed to yield any desired product with functionalization at the 7-position, but rather resulted in recovering the starting material 3-aminoindazole. 

### 2.1. Synthesis of 3-Bromo-2,6-dichlorobenzonitrile

**Scheme 2:**
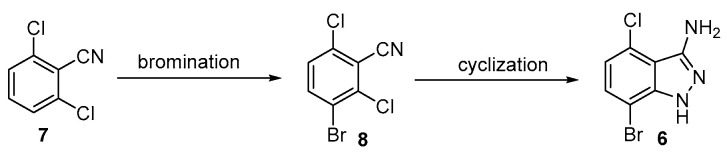
Synthesis of indazole **6** through regioselective cyclization of **8**.

As an alternative approach, it was hypothesized that the bromination of 2,6-dichlorobenzonitrile could provide compound **8** and this derivative could afford the desired 3-aminoindazole **6** (Scheme 2). 3-Bromo-2,6-dichlorobenzonitrile **8** was initially prepared according to a reported method in which the treatment of **7** with potassium bromate and sulfuric acid afforded **8** in a ~60% yield after column purification [14]. Unfortunately, this bromination method possessed potential safety issues as it was an extremely exothermic reaction. The conversion was also accompanied by side reactions, such as over-bromination and the hydrolysis of the cyano group. Decreasing the reaction temperature to 0 °C and −10 °C resulted in lower conversion with similarly low product purity. 

To identify more practical and safer bromination conditions to prepare 3-aminoindazole **8**, alternative brominating reagents and conditions were explored (Table 1). Two commonly used brominating reagents, Br_2_ and NBS, were investigated. Reactions with bromine resulted in the hydration of the cyano group to an amide. More hydrolyzed side products were observed under elevated temperatures (Table 1, entries 1–3). N-Bromosuccinimide (NBS) was identified as the optimal brominating reagent for this transformation. The initial reaction of **7** with 1.2 equivalents of NBS at 25 °C afforded product **8** in 75 A%, along with 12 A% of **7** and 12 A% of dibrominated product **8a** detected by GCMS total ion chromatography (TIC) (Table 1, entry 4). With an increase in the reaction temperature, the hydration of the nitrile group and over-bromination dominated (Table 1, entries 5–6). By decreasing the equivalents of NBS to 1.07 (25 °C), the reaction furnished **8** in >93 A% without the hydration of the nitrile group or over-bromination (Table 1, entry 7). An investigation of the equivalents and concentration of H_2_SO_4_ identified that 10–11 equivalents of 96% H_2_SO_4_ was optimal for the bromination (Table 1, entries 8–12). The use of 11 equivalents of 96% H_2_SO_4_ gave only 1 A% more **8**, compared to the use of 10 equivalents; thus, 10 equivalents of 96% H_2_SO_4_ was used for scale-up. Notably, swapping H_2_SO_4_ with other acids, such as TFA or acetic acid, resulted in no reaction (Table 1, entries 13–14). As a result, the optimal bromination conditions were identified for scale-up (NBS (1.07 eq.), 96% H_2_SO_4_ (10 eq.), 25 °C, 18 h). The optimized conditions were demonstrated in hundred-gram scale (25–300 g) reactions. After the completion of the reaction, the product mixture was poured into 15 volumes of ice-cold water and the resulting precipitate was collected by filtration. The filter cake was washed with 3 volumes of ethyl acetate to obtain the desired **8** in a 75–80% isolated yield with 95–96% purity (qNMR) (Table 1, entries 15–17).

### 2.2. Synthesis of 7-Bromo-4-chloro-1H-indazol-3-amine 

With 3-bromo-2,6-dichlorobenzonitrile (**8**) in hand, cyclization with hydrazine hydrate was investigated (Table 2). As shown in Table 2, a variety of solvents, such as aprotic polar solvents, protic solvents, basic solvents, and other organic solvents, were screened in the cyclization chemistry. Cyclization in aprotic polar solvents, such as NMP and DMSO, was performed smoothly under mild conditions (60 °C, 2 eq. of hydrazine hydrate and 1.2 eq. of NaOAc) [15]. Unfortunately, both chloro groups of **8** reacted with hydrazine without preference, resulting in a ~50:50 ratio of **6** and **12** (Table 2, entries 1–2). The ratio of **6**:**12** was improved to 65:35 when switching the solvent to ethanol and further improved to 70:30 with IPA as a solvent (Table 2, entries 3–4). In order to achieve >95% conversion of **8**, an elevated reaction temperature (95 °C) and excess hydrazine was needed. However, these promising results inspired our further solvent screening for better regioselectivity. Among all other solvents screened, DIPEA and 2-MeTHF were identified to afford a higher ratio of the desired regioisomer (Table 2, entries 5–9). For further optimization, 2-MeTHF was selected because it would be easily recycled, as it is water-immiscible. The equivalents of hydrazine were investigated to obtain optimal conditions for this transformation. It was found that 4 equivalents of hydrazine hydrate afforded >98% conversion of compound **8** (Table 2, entries 10–11). In addition, a solvent volume screen showed that 5 V of 2-MeTHF afforded >99% conversion with a 70:30 ratio of **6**/**12** (Table 2, entry 12). As a result, the optimized conditions for cyclization were identified for scale-up (hydrazine hydrate (4 eq.), NaOAc (1.2 eq.), 2-MeTHF (5 V), 95 °C).

Parenthetically, the transformation in 2-MeTHF needs an internal temperature of >90 °C to obtain full conversion; thus, a pressure reactor was utilized for scale-up. A mixture of **8** (20 g), NaOAc (1.2 eq.), and 4 eq. of hydrazine hydrate in 2-MeTHF (5 V) was stirred in a Parr reactor at 95 °C (internal temperature), affording >95% conversion after 18 h. It is worth mentioning that Kruger and coworkers found that the addition of sodium acetate is needed to mitigate the safety concerns regarding the utilization of hydrazine hydrate in scale [15]. The sodium acetate may quench the resulting HCl during cyclization, which suppresses the possible formation of high-energetic hydrazine HCl conjugates. Although the two isomers could be separated by column chromatography, a more scalable purification method by recrystallization to afford the desired isomer was identified. After screening a variety of solvents, a binary solvent of MeOH/H_2_O (80/20, *v*/*v*) was found as optimal for the recrystallization. For instance, a mixture of regioisomers (molar ratio of **6**/**12**: 70/30) was dissolved in MeOH/H_2_O (80/20, *v*/*v*) at 80 °C; after cooling to room temperature, the desired isomer **6** was obtained as a solid in an ~80% recovery yield with ~97% purity (qNMR). The protocol was successfully demonstrated with three batches in 20–80 g scale reactions. As shown in Table 3, in a Parr reactor, with 2-MeTHF as a solvent, the reaction of **8** and hydrazine hydrate at 95 °C afforded the crudes of the regioisomers (molar ratio of **6**/**12**: 70/30) in a quantitative yield. After treating the regioisomers with MeOH/H_2_O (80/20, *v*/*v*), the desired isomer **6** was obtained in a 50–56% isolated yield with 96–98 A% purity (GCMS) (Table 3, entries 1–3).

**Scheme 3:**
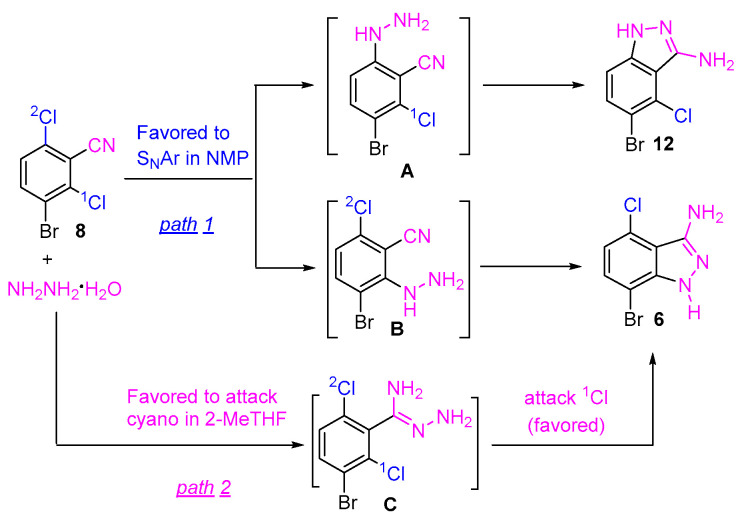
A plausible pathway for the formation of indazoles.

The subtle steric and electronic distinction of the two chlorine atoms might play an important role in the regioselective cyclization of **8** with hydrazine hydrate. However, the detailed mechanistic basis of the regioselectivity is unclear. It is known that the condensation reaction between 2,6-dichlorobenzonitrile and hydrazine hydrate can proceed with two possible pathways: (1) S_N_Ar occurs first, followed by an intramolecular cyano attack; or (2) the cyano attack occurs first, followed by an intramolecular S_N_Ar reaction [6,15]. The reaction of **8** and hydrazine might proceed with a similar pathway. As shown in Scheme 3, the indazole **6** could be formed through either hydrazine attacking the chloro first (S_N_Ar reaction), followed by intramolecular cyclization (path 1), or the hydrazine attacking the cyano group first, followed by intramolecular S_N_Ar cyclization (path 2). Both pathways afforded the indazole **6**. It is believed that pathway 1 might need less energy in the transformation of the intermediates to the indazole **6** (fast reaction rate, less hydrazine, and low reaction temperature), while pathway 2 might need higher energy to convert the intermediates to the indazole compound (long reaction time, more hydrazine, higher reaction temperature). Our experimental results indicated that the regioselective cyclization was highly solvent-dependent. We assumed that, in an aprotic polar solvent, such as NMP, pathway 1 was dominant and the fast reaction rate resulted in no selectivity. On the other hand, pathway 2 was dominant in 2-MeTHF. Presumably, the resulting intermediate **C** favors the intramolecular S_N_Ar reaction on ^1^Cl to afford the compound **6**, in which the inductive effect of the ortho bromine atom might play the role.

## 3. Experimental Section

### 3.1. General Method

Reagents and solvents were obtained from commercial suppliers and used as received, unless otherwise indicated. Where applicable, reactions were conducted in oven-dried (120 °C) glassware, which was assembled while hot and cooled to ambient temperature under an inert atmosphere. Reactors were pre-rinsed with reaction solvent and subjected to evacuation/back-fill cycles (3×) as necessary. Reactions were monitored by TLC (precoated silica gel 60 F254 plates, EMD Chemicals, Burlington, MA, USA), Agilent GCMS (Santa Clara, CA, USA), or crude ^1^H NMR. HRMS was recorded using a Perkin Elmer Axion 2 ToF MS (Waltham, MA, USA) in positive ionization mode with a scan range of 100–1000 *m*/*z*, flight tube voltage of 8 kV, spray voltage of 3.5 kV, and methanol as a solvent. TLC was visualized with UV light. The proton (^1^H NMR), carbon (^13^C NMR), and 2-DNMR spectra of the compounds were recorded on a Bruker Avance III HD Ascend 600 MHz spectrometer or 400 MHz spectrometer (Billerica, MA, USA). The NMR solvent used was DMSO-*d*_6_. The chemical shifts were reported in parts per million (ppm). Coupling constants *J* are reported in hertz (Hz). The abbreviations used to designate signal multiplicity were s, singlet; d, doublet.

### 3.2. Chemistry

#### 3.2.1. Preparation of 3-Bromo-2,6-dichlorobenzonitrile (**8**)

To a 5 L ChemRxnHub reactor (Chemglass Life Sciences LLC, Vineland, NJ, USA) at room temperature, 2,6-dichlorobenzonitrile (290.0 g, 1.68 mol) was added, followed by the addition of 96% sulfuric acid (10 eq., 0.92 L, 16.8 mol) with stirring at 0 °C. After the completion of the addition of sulfuric acid, the reaction mixture was stirred for 15 min to obtain a clear yellowish solution. *N*-bromosuccinimide (321 g, 1.07 eq., 1.8 mol) was added in portions over the course of 10 min at 0 °C. The reaction mixture was stirred at 25 °C for 18 h to afford a thick, pale-yellowish orange slurry. After the completion of the reaction (monitored by 1HNMR), the crude mixture was slowly transferred to ice water (2.9 L, 10 V). The slurry was stirred for 45 min and the resulting precipitates were collected by filtration. The solid cake was washed with water (500 mL × 5), dried under a house vacuum, and then washed with ethyl acetate (300 mL × 3). The solid was dried under a vacuum to obtain the product **8** (355 g, yield: 80%; purity by qNMR: 95%; purity by GCMS: 97%, containing 2% of dibromobenzonitrile **8a**). 

^1^H NMR (600 MHz, DMSO-*d*_6_) δ 8.11 (d, *J* = 8.8 Hz, 1H), 7.66 (d, *J* = 8.8 Hz, 1H). ^13^C NMR (151 MHz, DMSO-*d*_6_) δ 138.9, 137.3, 136.5, 129.8, 121.7, 114.5, 113.3. ^13^C NMR DEPT 135 (151 MHz, DMSO-*d*_6_) δ 138.9, 129.8. MS-EI (*m*/*z*): 251.

#### 3.2.2. Preparation of 7-Bromo-4-chloro-1*H*-indol-3-amine (**6**)

To a degassed 1 L Parr reactor was charged 3-bromo-2,6-dichlorobenzonitrile (80.0 g, 1 eq., 296 mmol), hydrazine hydrate (76 mL, 4 eq., 1.2 mol), and 2-MeTHF (5 V, 400 mL) at room temperature. The reaction mixture was heated at 105 °C and stirred for 18 h. After completion, the mixture was cooled to 25 °C. Water (3 V, 240 mL) was added and the mixture was extracted with ethyl acetate (300 mL × 3). The organic layer was combined and washed with brine (300 mL). The organic layer was separated and evaporated to dryness to afford a mixture of **6** and **12** (ratio: 70:29) with a quantitative mass (73 g crude). Methanol/water (4/1, *v*/*v*, ~20 V, 1.4 L) was added to the crude solid. The mixture was refluxed to afford a clear solution. The resulting solution was stirred at room temperature overnight. The white precipitates were filtered and washed with MTBE (20 mL × 4) to obtain the compound **6** (38 g, yield: 53%; purity by qNMR: 96%; purity by GCMS: 97%, containing 2% of dibromochloroindazole (*m*/*z*: 323)). 

^1^H NMR (600 MHz, DMSO-*d*_6_) δ 12.23 (s, 1H), 7.41 (d, *J* = 7.9 Hz, 1H), 6.85 (d, *J* = 7.9 Hz, 1H), 5.33 (s, 2H). ^13^C NMR (151 MHz, DMSO-*d*_6_) δ 149.1, 141.1, 129.5, 125.2, 119.1, 111.9, 101.0. ^13^C NMR DEPT 135 (151 MHz, DMSO-*d*_6_) δ 129.5, 119.1. HRMS (*m*/*z*): [M + H]^+^ calcd for C_7_H_5_BrClN_3_·H^+^: 247.9413 amu; found: 247.9412 amu.

For comparison, the undesired isomer **12** was purified by column chromatography (SiO_2_, ethyl acetate/heptanes = 10/90) to obtain the characterization data.

Compound **12**: ^1^H NMR (600 MHz, DMSO-*d*_6_) δ 12.0 (s, 1H), 7.45 (d, *J* = 8.8 Hz, 1H), 7.19 (d, *J* = 8.8 Hz, 1H), 5.27 (s, 2H). ^13^C NMR (151 MHz, DMSO-*d*_6_) δ 148.5, 141.8, 131.1, 125.7, 112.3, 110.9, 110.3. HRMS (*m*/*z*): [M + H]^+^ calcd for C_7_H_5_BrClN_3_·H^+^: 247.9413 amu; found: 247.9400 amu.

## 4. Conclusions

The practical synthesis of the heterocyclic compound 7-bromo-4-chloro-1*H*-indazol-3-amine has been developed from the readily available and inexpensive raw material 2,6-dichlorobenzonitrile. The two-step synthetic sequence utilizes a highly regioselective bromination and cyclization step to give an overall yield of 38–45% for the functionalized 3-aminoindazole (**6**). Mild bromination conditions (NBS/H_2_SO_4_) were identified, affording bromide **8** in a 76–81% yield from 2,6-dichlorobenzonitrile. The subsequent regioselective cyclization in 2-MeTHF afforded the desired 3-aminoindazole regioisomer in a 50–56% isolated yield with purity of up to 98%. The two-step protocol was demonstrated on hundred-gram scales and eliminates the need for column chromatography purification. This new chemistry provides a practical synthetic route to 7-bromo-4-chloro-1*H*-indazol-3-amine (**6**), a heterocyclic fragment used in the synthesis of the potent anti-HIV therapeutic, Lenacapavir.

## Figures and Tables

**Table 1:** Bromination of 2,6-dichlorobenzonitrile **7**.

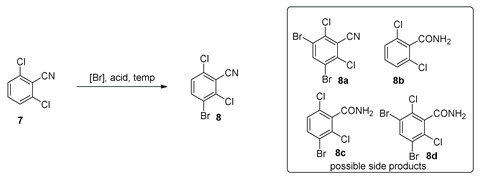									
Entry *^a^*	[Br] (eq.)	Acid (eq.)	Temp (°C)	GCMS Analysis (TIC A%) *^c^*					
				7	8	8a	8b	8c	8d
1 *^b^*	Br_2_ (2)	96% H_2_SO_4_ (10)	25	72	-	-	28	-	-
2 *^b^*	Br_2_ (2)	96% H_2_SO_4_ (10)	60	4	-	-	96	-	-
3 *^b^*	Br_2_ (2)	96% H_2_SO_4_ (10)	100	3	-	-	97	-	-
4 *^b^*	NBS (1.2)	96% H_2_SO_4_ (10)	25	12	75	12	-	-	-
5 *^b^*	NBS (1.2)	96% H_2_SO_4_ (10)	60	0.5	0.5	5	-	66	28
6 *^b^*	NBS (1.2)	96% H_2_SO_4_ (10)	100	-	-	4	-	61	32
7	NBS (1.07)	96% H_2_SO_4_ (10)	25	3	93	4	-	-	-
8	NBS (1.07)	96% H_2_SO_4_ (5)	25	21	70	9	-	-	-
9	NBS (1.07)	96% H_2_SO_4_ (9)	25	7	92	-	-	-	-
10	NBS (1.07)	96% H_2_SO_4_ (11)	25	2	94	4	-	-	-
11	NBS (1.07)	60% H_2_SO_4_ (10)	25	>95	-	-	-	-	-
12	NBS (1.07)	80% H_2_SO_4_ (10)	25	14	66	20	-	-	-
13	NBS (1.07)	TFA (10)	60	>95	-	-	-	-	-
14	NBS (1.07)	Acetic acid (10)	60	>95	-	-	-	-	-
15 *^d^*	NBS (1.07)	96% H_2_SO_4_ (10)	25	4	93	3	-	-	-
16 *^e^*	NBS (1.07)	96% H_2_SO_4_ (10)	25	5	91	4	-	-	-
17 *^f^*	NBS (1.07)	96% H_2_SO_4_ (10)	25	5	91	4	-	-	-

*^a^* All reactions were performed with 2,6-dichlorobenzonitrile (2 g) under the conditions shown in the table for 18 h, unless otherwise stated. *^b^* 0.1 g of 2,6-dichlorobenzonitrile. *^c^* All of these data are the IPC of crude reaction mixtures obtained by GCMS total ion chromatogram (TIC) and reported as A%, unless otherwise stated. *^d^* 25 g of 2,6-dichlorobenzonitrile; isolated yield: 76%, 96% purity by qNMR. *^e^* 125 g of 2,6-dichlorobenzonitrile; isolated yield: 81%, 96% purity by qNMR. *^f^* 290 g of 2,6-dichlorobenzonitrile, isolated yield: 80%, 95% purity by qNMR.

**Table 2:** Optimization of cyclization of **8**.

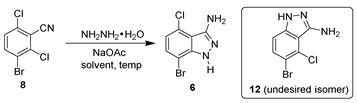					
Entry *^a^*	Solvent	Temp (°C)	Eq. of NH_2_NH_2_·H_2_O	Conversion (%) *^b^*	Ratio *^c^*
					6:12
1	NMP	60	2	100	~50:50
2	DMSO	60	2	100	~50:50
3	EtOH	95	10	100	65:35
4	IPA	95	10	100	70:30
5	DIPEA	95	10	100	75:25
6	Pyridine	95	10	100	68:32
7	THF	95	10	100	70:30
8	Diglyme	95	10	100	70:30
9	2-MeTHF	95	10	100	73:27
10	2-MeTHF	95	8	100	70:30
11	2-MeTHF	95	4	98	70:30
12 *^d^*	2-MeTHF	95	4	99	70:30

*^a^* All reactions were performed in heavy-wall pressure vessels with **8** (0.5 g) in the presence of NaOAc (1.2 eq.) under the conditions shown in the table for 18 h, unless otherwise stated; 10 solvent volumes were used (V) = mL/g of **8**. *^b^* Monitored by GCMS-TIC. *^c^* This was the crude reaction mixture ratio and monitored by ^1^HNMR. *^d^* 5 V of 2-MeTHF was used.

**Table 3:** Synthesis and purification of 7-bromo-4-chloro-1*H*-indazol-3-amine.

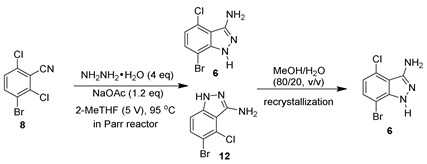						
Entry *^a^*	Reaction Scale	A% (GCMS (TIC)) of Crude *^b^*			6 after Crystallization *^c^*	
		8	12	6	Purity by GCMS (TIC A%)	Isolated Yield
1	20 g	2	28	70	97 *^d^*	50%
2	40 g	<1	29	70	98 *^e^*	56%
3	80 g	<1	29	70	96 *^f^*	53%

*^a^* All reactions were performed in a Parr reactor with **8** (20–80 g), hydrazine hydrate (4 eq.), NaOAc (1.2 eq.), 2-MeTHF (5 V), 95 °C (internal temperature), 18 h. *^b^* All of these crude mixtures were monitored by GCMS (TIC) and are reported as A%. *^c^* The crude was dissolved in MeOH/H_2_O (80/20, *v*/*v*) at 80 °C and then cooled to rt, collected by filtration, and washed with MTBE. The purity was measured by GCMS-TIC A%). *^d^* Containing ~2% of **12** and ~1% unknown impurity. *^e^* Containing ~2% of unknown impurity. *^f^* Containing ~2% of **12** and ~2% unknown impurity.

## Data Availability

The data presented in this study are available in the main text and the Supplementary Materials of this article.

## Supplementary Materials

The following supporting information can be downloaded at: https://www.mdpi.com/article/10.3390/molecules29122705/s1, GCMS method. Detailed procedure of synthesis of **6** from 3-bromo-6-chloro-2-fluorobenzonitrile; Detailed procedure of synthesis of **9** and **10**. Table S1: Recrystallization of 7-bromo-4-chloro-1*H*-indazol-3-amine **6**. Figure S1: ^1^HNMR of 3-bromo-2,6-dichlorobenzonitrile (**8**) in DMSO-*d*_6_ (600 MHz). Figure S2. ^13^CNMR of 3-bromo-2,6-dichlorobenzonitrile (**8**) in DMSO-*d*_6_. Figure S3. DEPT-135 of 3-bromo-2,6-dichlorobenzonitrile (**8**) in DMSO-*d*_6_. Figure S4. ^1^HNMR of 7-bromo-4-chloro-1*H*-indazole-3-amine (**6**) in DMSO-*d*_6_. Figure S5. ^13^CNMR of 7-bromo-4-chloro-1*H*-indazole-3-amine (**6**) in DMSO-*d*_6_. Figure S6. DEPT-135 of 7-bromo-4-chloro-1*H*-indazole-3-amine (**6**) in DMSO-*d*_6_. Figure S7. ^1^H-^1^H COSY of 7-bromo-4-chloro-1*H*-indazole-3-amine (**6**) in DMSO-*d*_6_. Figure S8. HMBC of 7-bromo-4-chloro-1*H*-indazole-3-amine (**6**) in DMSO-*d*_6_. Figure S9. ^1^HNMR of 5-bromo-4-chloro-1*H*-indazole-3-amine (**12**) in DMSO-*d*_6_. Figure S10. ^13^CNMR of 5-bromo-4-chloro-1*H*-indazole-3-amine (**12**) in DMSO-*d*_6_. Figure S11. ^1^H-^1^H COSY of 5-bromo-4-chloro-1H-indazole-3-amine (**12**) in DMSO-*d*_6_. Figure S12. HSQC of 5-bromo-4-chloro-1*H*-indazole-3-amine (**12**) in DMSO-*d*_6_. Figure S13. ^1^HNMR of 4-chloro-1*H*-indazole-3-amine (**9**) in DMSO-*d*_6_. Figure S14. ^1^HNMR of **10a** in DMSO-*d*_6_. Figure S15. ^1^HNMR of **10** in DMSO-*d*_6_.
