# Supplementary material for: Practical Synthesis of 7-Bromo-4-chloro-1H-indazol-3-amine: An Important Intermediate to Lenacapavir
Source: Molecules. 2024 Jun 7;29(12):2705. doi: 10.3390/molecules29122705 (PMC11206596; doi:10.3390/molecules29122705)
Supplement: Supplementary file 1 [file molecules-29-02705-s001.zip › molecules-3016980-supplementary.pdf]

Supporting Information

Practical Synthesis of 7-Bromo-4-chloro-1H-indazol-3-amine: An Important Intermediate to Lenacapavir

*Naeem Asad,[†] Michael Lyons,[†] Shirley Muniz Machado Rodrigues, Justina M. Burns, Thomas D. Roper, G. Michael Laidlaw, Saeed Ahmad, B. Frank Gupton, Douglas Klumpp, Limei Jin**

Medicines for All Institute, Virginia Commonwealth University, Richmond, VA 23284-3068, USA.

[†]These authors contributed equally

Email: jinl3@vcu.edu

Table of Contents

General Method 2

GC-MS Method 2

Experimental procedure 6

Preparation of 7-bromo-4-chloro-1H-indazol-3-amine (6) as a standard based on literature method 6

Preparation of 3-bromo-2,6-dichlorobenzonitrile (8) 6

Preparation of 7-bromo-4-chloro-1H-indol-3-amine (6) 7

Preparation of 4-chloro-1H-indazol-3-amine (9) 7

Preparation of 1-(3-(bis(trimethylsilyl)amino)-4-chloro-1H-indazol-1-yl)-2,2-dimethylpropan-1-one (10) 8

Table S1. Recrystallization of 7-bromo-4-chloro-1H-indazol-3-amine 6. 9

NMR Spectra 10

3-bromo-2,6-dichlorobenzonitrile (8) 10

7-bromo-4-chloro-1H-indazol-3-amine (6) 12

5-bromo-4-chloro-1H-indazol-3-amine (12) 15

4-chloro-1H-indazol-3-amine (9) 18

1-(3-(bis(trimethylsilyl)amino)-4-chloro-1H-indazol-1-yl)-2,2-dimethylpropan-1-one (10) 19

General Method

Reagents and solvents were obtained from commercial suppliers and used as received unless otherwise indicated. Where applicable, reactions were conducted in oven-dried (120°C) glassware, which was assembled while hot, and cooled to ambient temperature under an inert atmosphere. Reactors were pre-rinsed with reaction solvent and subjected to evacuation/back-fill cycles (3×) as necessary. Reactions were monitored by TLC (precoated silica gel 60 F254 plates, EMD Chemicals), Agilent GCMS or crude ^{1}H NMR. HRMS was recorded using Perkin Elmer Axion 2 ToF MS, ionization mode: positive with scan range: 100 - 1000 m/z, flight tube voltage: 8 kV, spray voltage: 3.5 kV, solvent: methanol. TLC was visualized with UV light. The proton (^{1}H NMR), carbon (^{13}C NMR) and 2-DNMR spectra of the compounds were recorded on Bruker Avance III HD Ascend 600 MHz spectrometer. The NMR solvents used were DMSO-d_6, $CDCl_3$ and CD_3OD. The chemical shifts were reported in parts per million (ppm). Coupling constants J are reported in hertz (Hz). The abbreviations used to designate signal multiplicity were: s, singlet; d, doublet; t, triplet; q, quartet, p, pentet; dd, doublet of doublets; ddd, doublet of doublet of doublets; dt, double of triplets; ddt, doublet of doublet of triplets; m, multiplet; br, broad.

GC-MS Method

Formation of product and side-products was monitored via GC-MS (Agilent 6890/8890 GC-5977 MSD). An Agilent J&W HP-5ms GC Column, 30 m, 0.25 mm, 0.25 µm, 7 inch cage was used for analysis. The inlet was set to 250 °C. A split ratio of 100:1 was used with an injection volume of 1.0 µL. The column flow rate was 1.4 mL/min with ultra high purity helium as the carrier gas and an inlet pressure of 7.87 psi. The oven was initially set to 50 °C for 3 minutes, linearly ramped to 250 °C at 25 °C/min and held for 3 minutes. The column temperature was ramped a final time at 25 °C/min to 300 °C which was held for 3 min.

Structures & IDs:

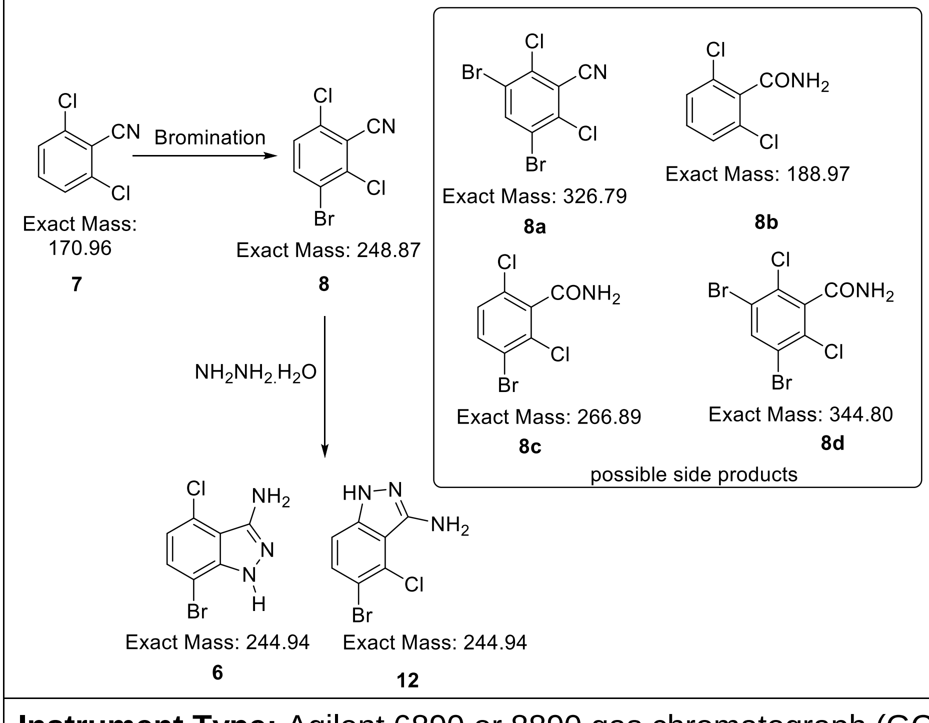

Instrument Type: Agilent 6890 or 8890 gas chromatograph (GC) with a 5977 mass spectrometer detector (MSD)

Conditions:

Column: J&W HP-5ms GC Column, 30 m, 0.25 mm, 0.25 µm, 7 inch cage

Inlet Pressure: 11.747 psi | Split Ratio: 100:1 | Split Flow: 140 mL/min

Column flow: 1.4 mL/min | Injection Temp: 250ºC | Injection volume: 1 µL

Total Flow: 144.4 mL/min | Solvent Delay: 3 min | Runtime: 19 min

Temperature Program:

Time (min)	Temp (˚C)	Ramp (˚C/min)	Hold (min)
0	50	-	3
-	250	25	3
-	300	25	3

MS Parameters:

Transfer Line Temp (˚C)	250
Source Temp (˚C)	230
Quad Temp (˚C)	150
Electron Energy (eV)	70
Mass Range	40-1000

Sample preparation: Samples are prepared at ~0.2 mg/mL in acetonitrile

Retention Times

Compound	Primary *m/z*	Secondary *m/z*	Time (min)
7	171	136	8.4
8	251	170	9.7
8a	327	250	10.8
6	247	218	11.1
12	247	218	12.0

Notes: **Notes:** For samples submitted in DMSO, use the 6 min delay method as DMSO comes out around 4.5 minutes which will swamp the GCMS. The 6 and 12 are isomers that have different solubilities between their desired and undesired forms so methanol should be avoided to prevent crashing out the undesired isomers.

Representative Chromatogram(s) (attach additional chromatograms and spectra as needed)

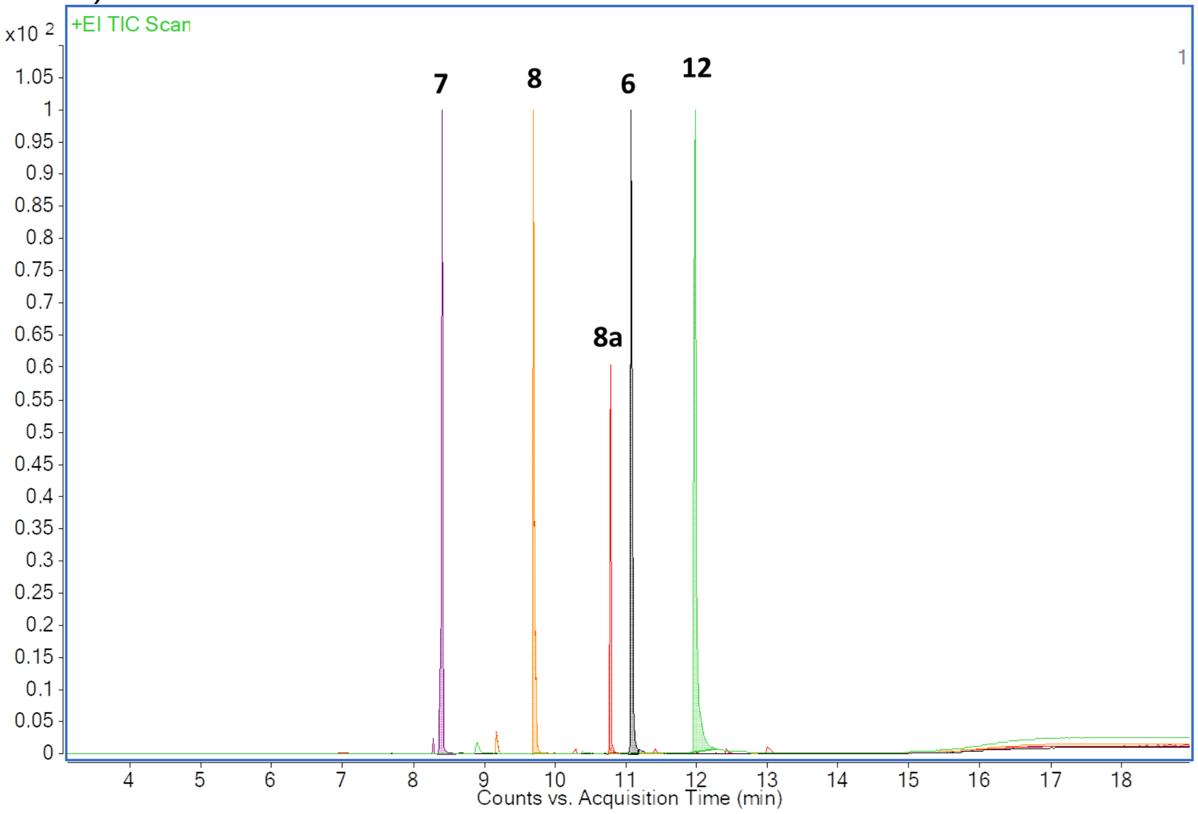

Mass spectra:

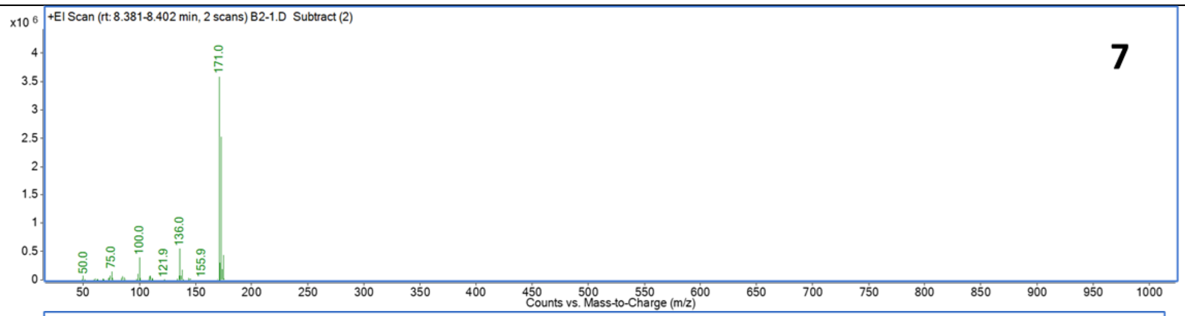

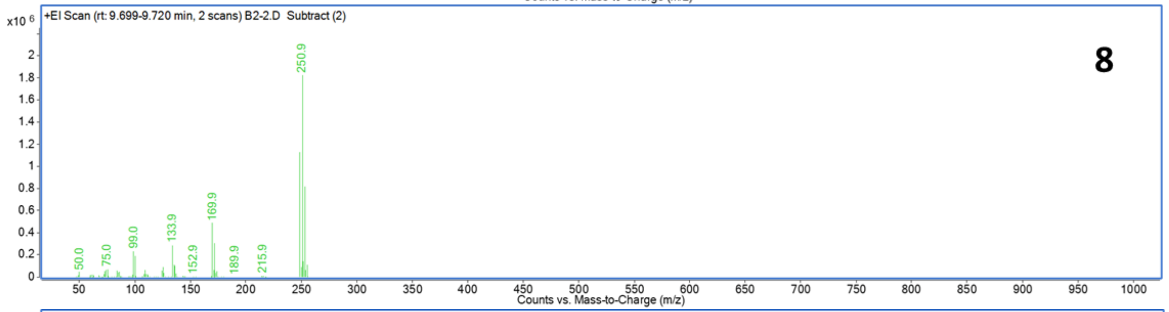

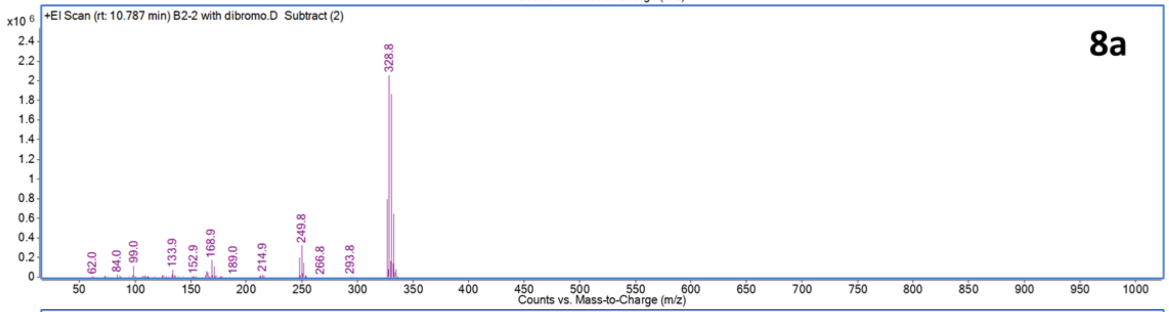

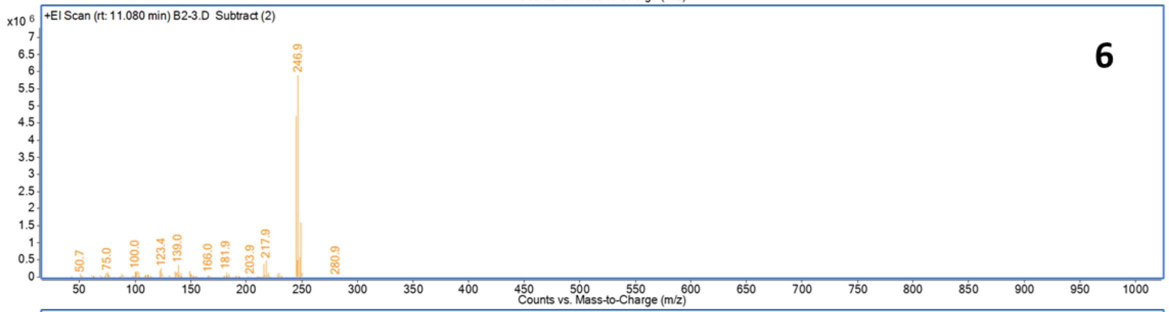

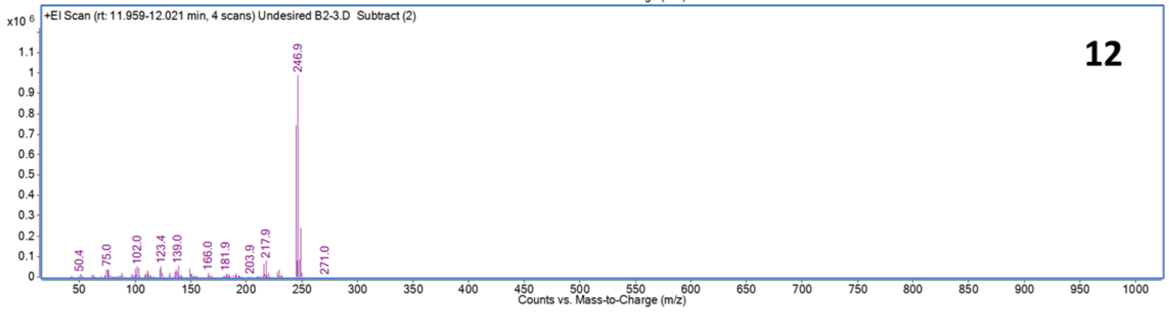

Calibration Curves:

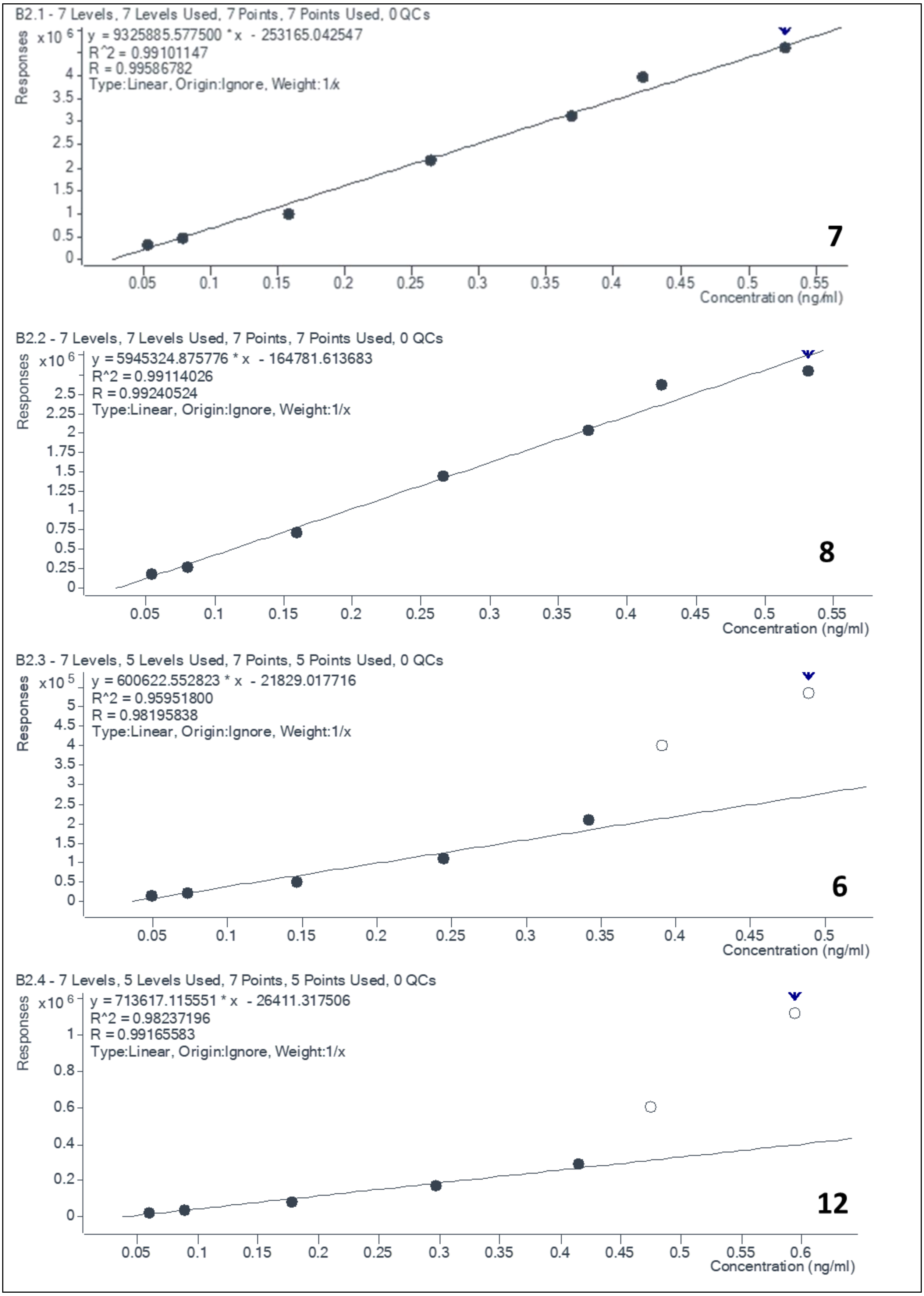
B2.1 - 7 Levels, 7 Levels Used, 7 Points, 7 Points Used, 0 QCs
y = 9325885.577500 * x - 253165.042547
R^2 = 0.99101147
R = 0.99586782
Type:Linear, Origin:Ignore, Weight:1/x
Responses
x10 6
Concentration (ng/ml)
7
B2.2 - 7 Levels, 7 Levels Used, 7 Points, 7 Points Used, 0 QCs
y = 5945324.875776 * x - 164781.613683
R^2 = 0.99114026
R = 0.99240524
Type:Linear, Origin:Ignore, Weight:1/x
Responses
x10 6
Concentration (ng/ml)
8
B2.3 - 7 Levels, 5 Levels Used, 7 Points, 5 Points Used, 0 QCs
y = 600622.552823 * x - 21829.017716
R^2 = 0.95951800
R = 0.98195838
Type:Linear, Origin:Ignore, Weight:1/x
Responses
x10 5
Concentration (ng/ml)
6
B2.4 - 7 Levels, 5 Levels Used, 7 Points, 5 Points Used, 0 QCs
y = 713617.115551 * x - 26411.317506
R^2 = 0.98237196
R = 0.99165583
Type:Linear, Origin:Ignore, Weight:1/x
Responses
x10 6
Concentration (ng/ml)
12

Experimental procedure

Preparation of 7-bromo-4-chloro-1H-indazol-3-amine (6) as a standard based on literature method [1]

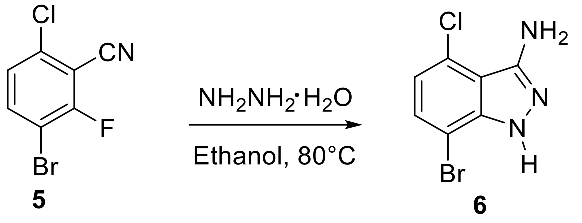

A mixture of 3-bromo-6-chloro-2-fluorobenzonitrile (250 mg), ethanol (2.0 mL) and hydrazine hydrate (0.35 mL; 5 eq) was stirred at 80 °C for 1 h. After completion, the solution was allowed to cool to 45 °C and water was added slowly to produce a white precipitate. Following the addition of water, the mixture was stirred for 30 minutes. The solids were isolated via filtration. The solids were washed with water and then dried under vacuum at 45 °C to afford the desired compound 7-bromo-4-chloro-lH-indazol-3-amine (**6**) in 87% yield and 99.8% purity (GCMS-TIC).

^{1}H NMR (600 MHz, DMSO-d6) δ 12.23 (s, 1H), 7.41 (d, J = 7.9 Hz, 1H), 6.85 (d, J = 7.9 Hz, 1H), 5.33 (s, 2H). ^{13}C NMR (151 MHz, DMSO-d6) δ 149.1, 141.1, 129.5, 125.2, 119.1, 111.9, 101.0.

MS-EI (m/z): 245 and 247.

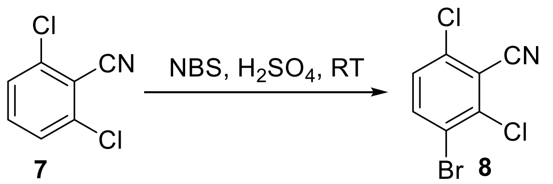

Preparation of 3-bromo-2,6-dichlorobenzonitrile (8)

To a 5 L ChemRxnHub reactor at room temperature, 2,6-dichlorobenzonitrile (290.0 g, 1.68 mol) was added followed by addition of 96% sulfuric acid (10 eq., 0.92 L, 16.8 mol) with stirring at 0°C. After completion of addition of sulfuric acid the reaction mixture was stirred for 15 minutes to obtain a clear yellowish solution. N-bromosuccinimide (321 g, 1.07 eq., 1.8 mol) was added in portions over the course of 10 minutes at 0°C. The reaction mixture was stirred at 25 °C for 18h to afford a thick, pale yellowish orange slurry. After completion of the reaction (monitored by ^{1}HNMR), the crude mixture was slowly transferred to ice-water (2.9 L, 10V). The slurry was stirred for 45 minutes and the resulting precipitates were collected by filtration. The solid cake was washed with water (500 mL ×5), dried under house vacuum and then washed with ethyl acetate (300 mL × 3). The solid was dried under vacuum to obtain the product (355 g, yield: 80%; purity by qNMR: 95%; purity by GCMS: 97%, containing 2% of dibromobenzonitrile **8a**).

^{1}H NMR (600 MHz, DMSO-d6) δ 8.11 (d, J = 8.8 Hz, 1H), 7.66 (d, J = 8.8 Hz, 1H).

^{13}C NMR (151 MHz, DMSO-d6) δ 138.9, 137.3, 136.5, 129.8, 121.7, 114.5, 113.3.

^{13}C NMR DEPT 135 (151 MHz, DMSO-d6) δ 138.9, 129.8.

MS-EI (m/z): 251.

---

[1] Link, J. O. et al. *Nature* **2020**, *584*, 614–618.

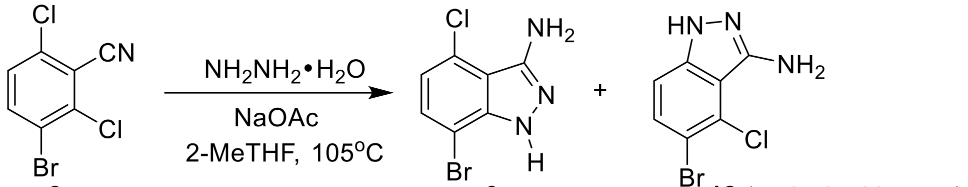

8 **6** **12** (undesired isomer)

Preparation of 7-bromo-4-chloro-1H-indol-3-amine (6)

To a degassed 1L Parr reactor was charged 3-bromo-2,6-dichlorobenzonitrile (80.0 g, 1 eq., 296 mmol), hydrazine hydrate (76 mL, 4 eq., 1.2 mol) and 2-MeTHF (5 V, 400 mL) at room temperature. The reaction mixture was heated at 105°C and stirred for 18h. After completion, the mixture was cooled to 25 °C. Water (3V, 240 mL) was added and the mixture was extracted with ethyl acetate (300 mL ×3). The organic layer was combined and washed with brine (300 mL). The organic layer was separated and evaporated to dryness to afford a mixture of **6** and **12** (ratio: 70:29) with a quantitative mass (73 g crude). Methanol/water (4/1, v/v, ~20V, 1.4 L) was added to the crude solid. The mixture was refluxed to afford a clear solution. The resulting solution was stirred at room temperature overnight. The white precipitates were filtered and washed with MTBE (20 mL × 4) to obtain the compound **6** (38g, yield: 53%; purity by qNMR: 96%; purity by GCMS: 97%, containing 2% of dibromochloroindazole (m/z: 323)).

^{1}H NMR (600 MHz, DMSO-d6) δ 12.23 (s, 1H), 7.41 (d, J = 7.9 Hz, 1H), 6.85 (d, J = 7.9 Hz, 1H), 5.33 (s, 2H). ^{13}C NMR (151 MHz, DMSO-d6) δ 149.1, 141.1, 129.5, 125.2, 119.1, 111.9, 101.0. ^{13}C NMR DEPT 135 (151 MHz, DMSO-d6) δ 129.5, 119.1. HRMS (m/z): $[M+H]^+$ calcd for $C_7H_6BrClN_3^+$: 247.9413 amu; found: 247.9412 amu.

For comparison, the undesired isomer **12** was purified by column chromatography (SiO_2, ethyl acetate/heptanes = 10/90) to obtain the characterization data.

Compound **12**: ^{1}H NMR (600 MHz, DMSO-d6) δ 12.0 (s, 1H), 7.45 (d, J = 8.8 Hz, 1H), 7.19 (d, J = 8.8 Hz, 1H), 5.27 (s, 2H). ^{13}C NMR (151 MHz, DMSO-d6) δ 148.5, 141.8, 131.1, 125.7, 112.3, 110.9, 110.3. HRMS (m/z): $[M+H]^+$ calcd for $C_7H_6BrClN_3^+$: 247.9413 amu; found: 247.9400 amu.

Preparation of 4-chloro-1H-indazol-3-amine (9)[2]

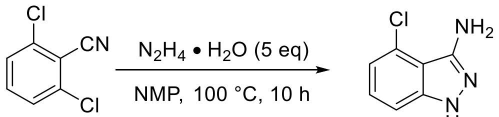

7 **9**

To a round-bottom flask (1 L) equipped with a magnetic stirring bar were added 2,6-dichlorobenzonitrile (40 g, 232.5 mmol) and NMP (400 mL). Hydrazine hydrate (59.39 g, 5 eq, 1.16 mol, 57.55 mL) was added dropwise at 25 °C. The mixture was stirred at 100 °C for 10 h. Water (600 mL) was added and the mixture was extracted with EtOAc (500 mL × 4). The combined organic layer was washed with brine (2 L × 3), dried over Na_2SO_4 and concentrated under reduced pressure to afford **9** (38.8 g, 90% purity by qNMR, 90% isolated yield) a yellow solid containing ~10% NMP. The crude was used for the next step without further purification.

^{1}H NMR (400 MHz, DMSO-d6) δ = 11.82 (br s, 1H), 7.22 - 7.17 (m, 2H), 6.89 (dd, *J* = 1.2, 6.8 Hz, 1H), 5.17 (s, 2H).

---

[2] Kruger, A. W. et al. *Org. Process Res. Dev.* **2009**, *13*, 1419–1425.

Preparation of 1-(3-(bis(trimethylsilyl)amino)-4-chloro-1H-indazol-1-yl)-2,2-dimethylpropan-1-one (10)

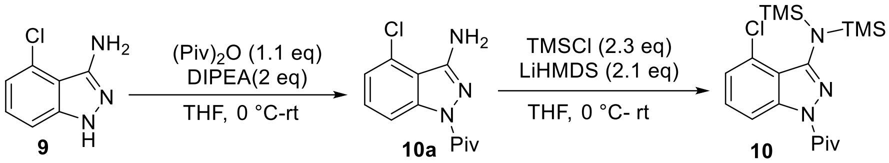

To a three-necked flask (250 mL) equipped with a thermometer and a magnetic stirring bar were added 4-chloro-1H-indazol-3-amine (**9**, 5 g, 26.85 mmol), DIPEA (6.94 g, 53.70 mmol, 9.35 mL, 2 eq) and THF (100 mL). The mixture was cooled to 0°C and to the mixture was added pivalic anhydride (5.50 g, 29.54 mmol, 5.99 mL, 1.1 eq) at 0 ^{o}C. The reaction mixture was stirred at 25 °C for 12 h. After completion of reaction, the mixture was evaporated to dryness. The residue was purified by column chromatography (SiO_2, PE/EtOAc =1/0 to 20/ 1) to afford **10a** as a white solid (4.8 g, isolated yield: 71%).

Compound **10a**:

^{1}H NMR (400 MHz, DMSO-d6) δ = 8.26 (d, *J* = 8.4 Hz, 1H), 7.51 (t, *J* = 8.0Hz, 1H), 7.33 (d, *J* = 7.6 Hz, 1H), 6.12 (s, 2H), 1.46 (s, 9H).

MS-ESI (*m/z*) ($M+H^+$): 252.1 amu.

To a three-necked flask (100 mL) equipped with a thermometer and a magnetic stirring bar were added 1-(3-amino-4-chloro-indazol-1-yl)-2,2-dimethyl-propan-1-one (**10a**, 500 mg, 1.99 mmol, 1 eq) and THF (8 mL). The mixture was cooled to 0°C. TMSCl (496.35 mg, 4.57 mmol, 579.84 µL, 2.3 eq) was added dropwise followed by LiHMDS (1 M, 4.17 mL, 2.1 eq) at 0 ^{o}C under N_2. The resulting mixture was stirred at 0 ^{o}C for 2h under N_2. The crude **10** was used for the next lithiation followed by bromination or borylation, but no reaction occurred.

Compound **10**:

^{1}H NMR (400 MHz, DMSO-d6) δ = 8.21 (d, *J* = 8.0 Hz, 1H), 7.45 (t, *J* = 8.0Hz, 1H), 7.30 (d, *J* = 7.2 Hz, 1H), 1.39 (s, 9H), 0.01 (s, 9H), 0.00 (s, 9H).

Table S1. Recrystallization of 7-bromo-4-chloro-1H-indazol-3-amine **6.**

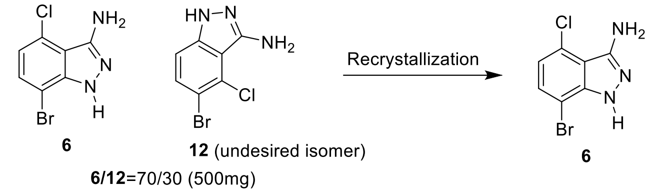

Entry	Conditions	Purity of **6** by qNMR	Recovery yield of **6**
1	MeOH (12V), rt	96%	15%
2	1,2-dimethoxyethane (2V), 80°C-rt	95%	20%
3	1,4-dioxane (10V), rt	70%	NM
4	THF (10V), rt	70%	NM
5	2-MeTHF (10V), rt	80%	NM
6	2-methylbutan-2-ol (10V), rt	70%	NM
7	Toluene (10V), rt	70%	NM
8	iPrOAc (10V), rt	85%	NM
9	DCE (10V), rt	80%	NM
10	EtOAc (10V), rt	85%	NM
11	CH3CN (10V), rt	80%	NM
12	EtOH (two drops of NH_4OH) (5V), 80°C-rt	70%	NM
13	DME (two drops of NH_4OH) (2V), 80°C-rt	70%	NM
14	MeOH (0.06eq HCl) (4V), 80°C-rt	70%	NM
15	H2O (1eq HCl) (30V), 80°C-rt	70%	NM
16	MeOH:H_2O 80:20 (31 V), 80°C-rt	95%	70%
17	**MeOH:H_2O 80:20 (20 V), 80°C-0°C**	**97%**	**70%**
18	MeOH:H_2O 60:40 (69 V), 80°C-0°C	96%	78%
19	MeOH:H_2O 40:60 (40 V), 80°C-0°C	87%	58%
20	MeOH:H_2O 20:80 (40 V), 80°C-0°C	80%	84%

Solvent volume (V) = mL/g. NM: not measured.

NMR Spectra

3-bromo-2,6-dichlorobenzonitrile (8)

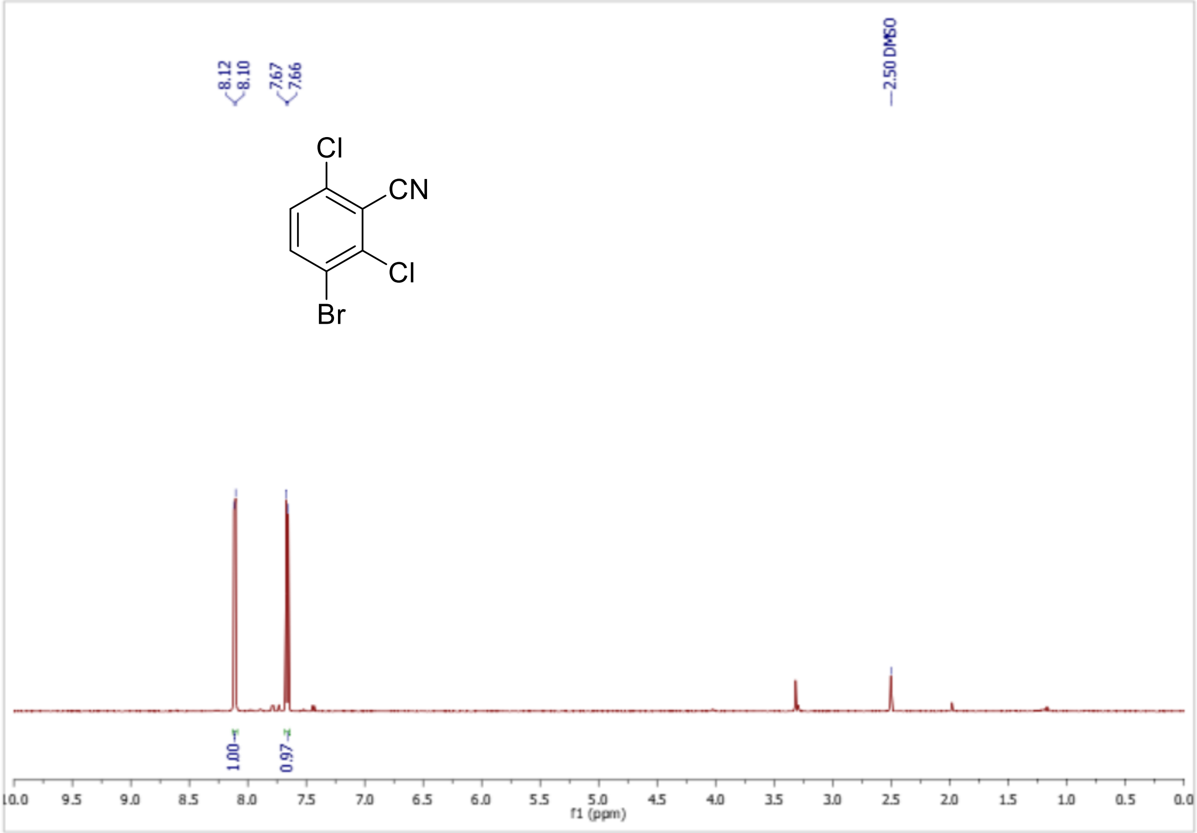

Figure S1. ^{1}HNMR of 3-bromo-2,6-dichlorobenzonitrile (**8**) in DMSO-d6.

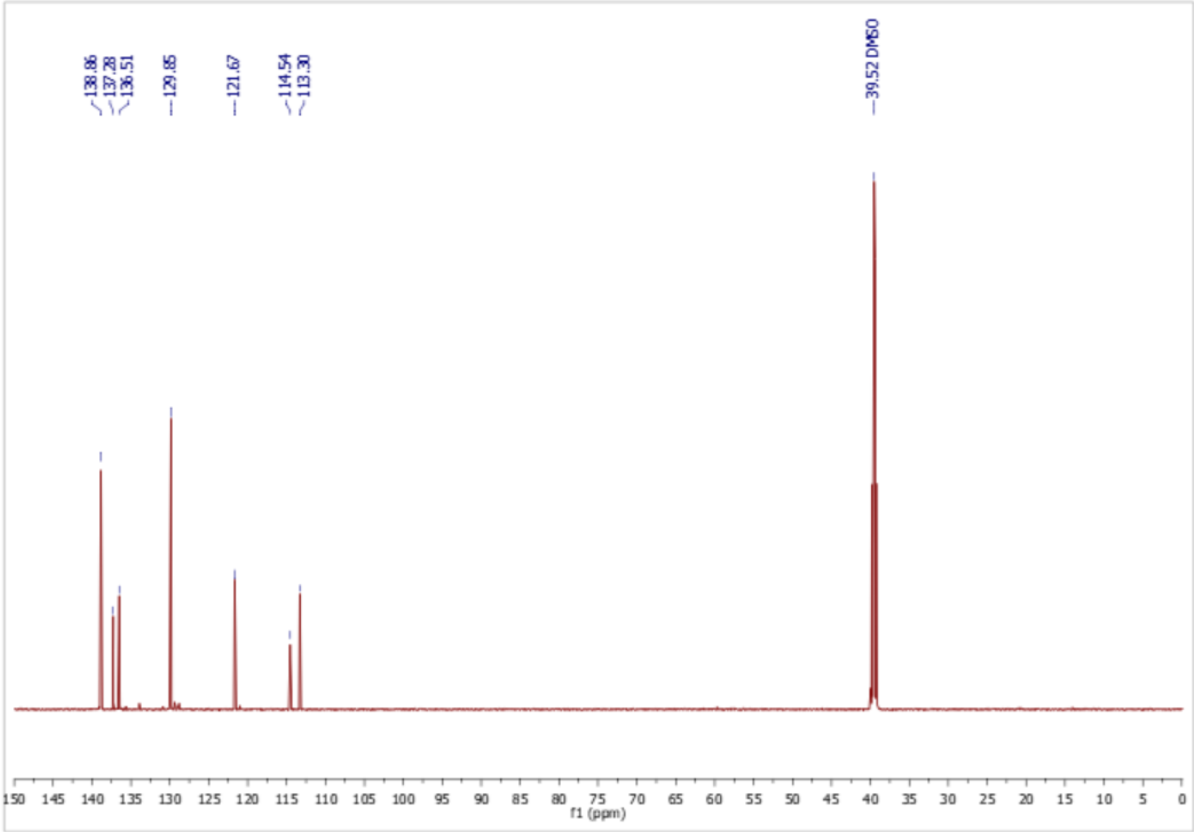

Figure S2. ^{13}CNMR of 3-bromo-2,6-dichlorobenzonitrile (**8**) in DMSO-d6.

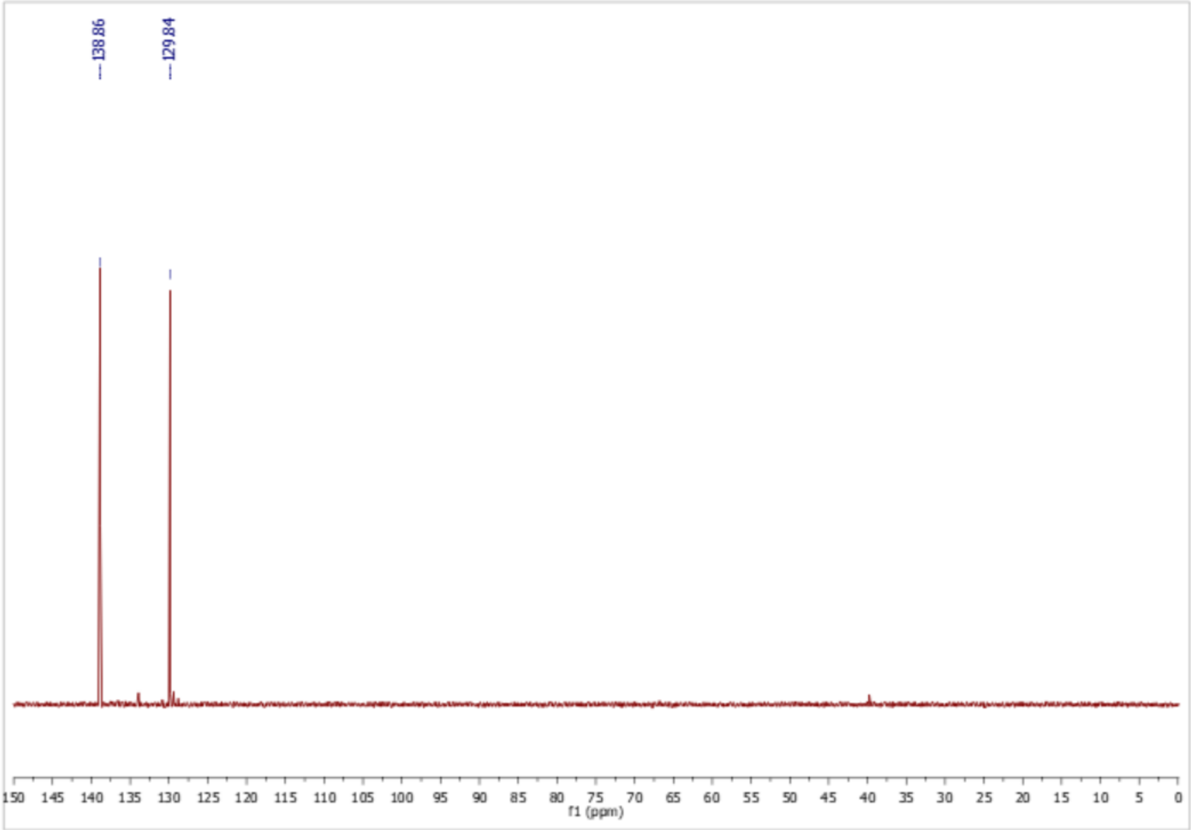

Figure S3. DEPT-135 of 3-bromo-2,6-dichlorobenzonitrile (**8**) in DMSO-d6.

7-bromo-4-chloro-1H-indazol-3-amine (6)

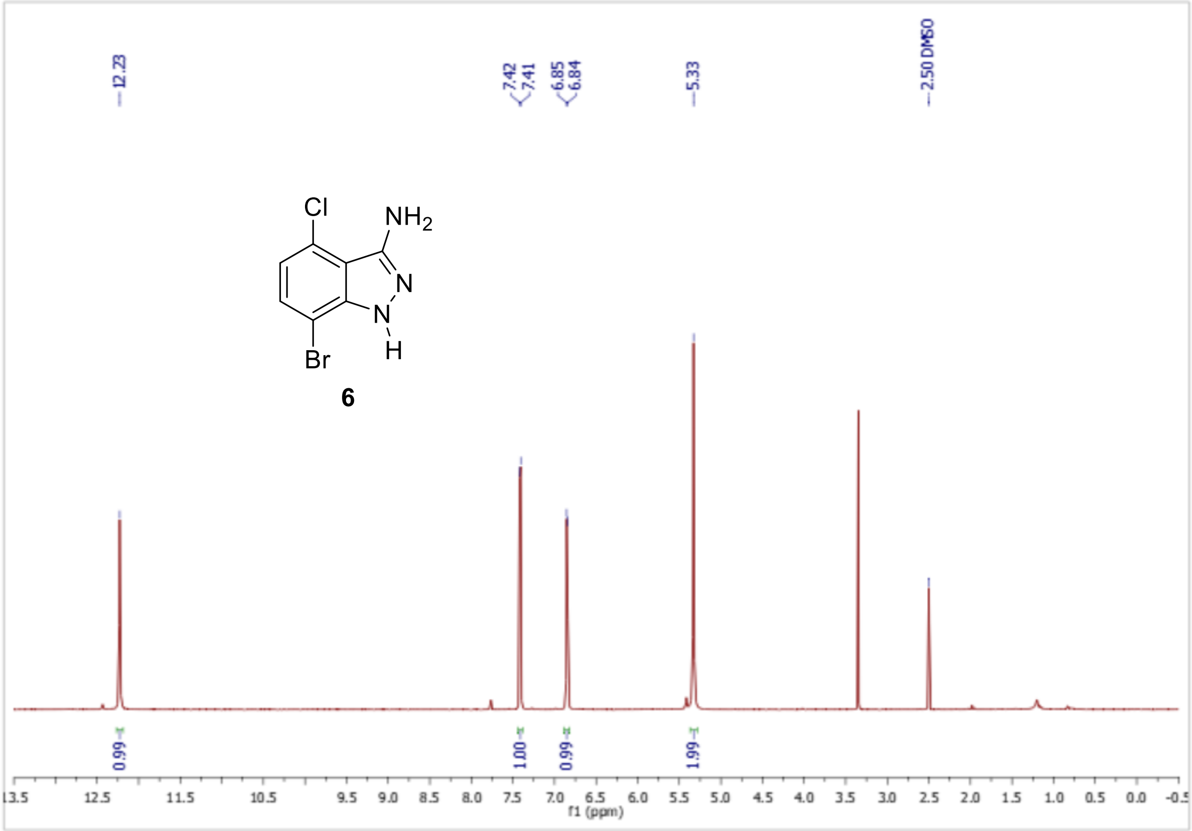

Figure S4. ^{1}HNMR of 7-bromo-4-chloro-1H-indazole-3-amine (**6**) in DMSO-d6.

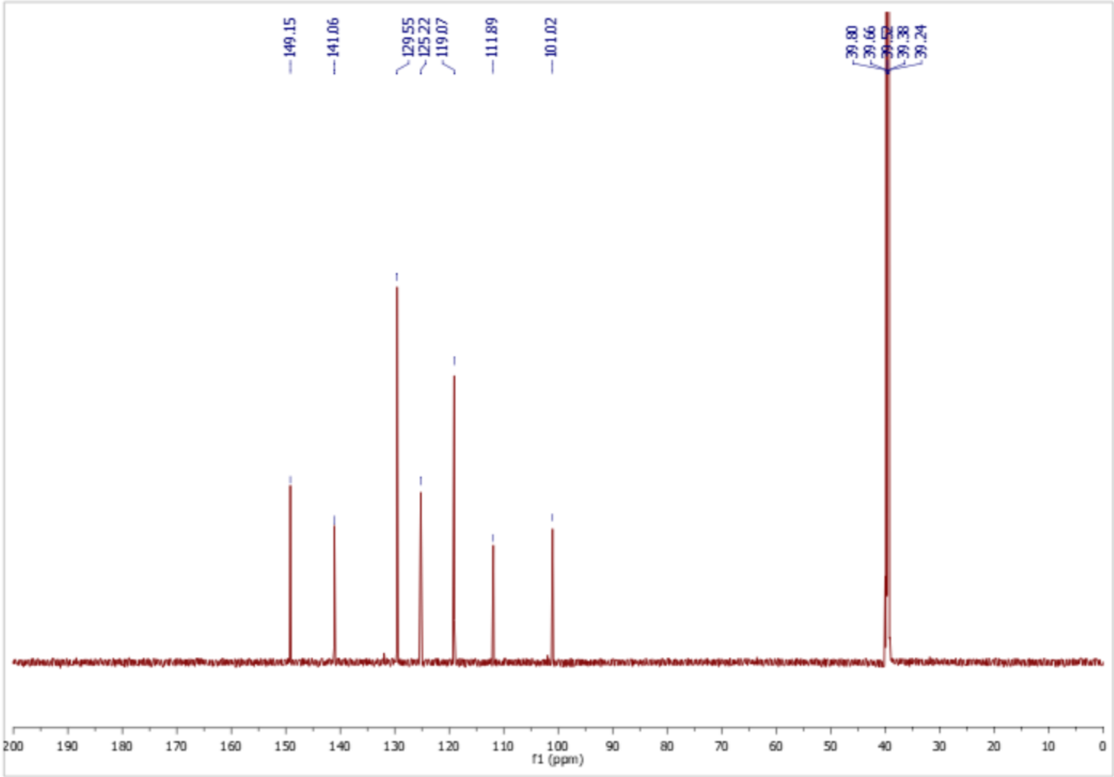

Figure S5. ^{13}CNMR of 7-bromo-4-chloro-1H-indazole-3-amine (**6**) in DMSO-d6.

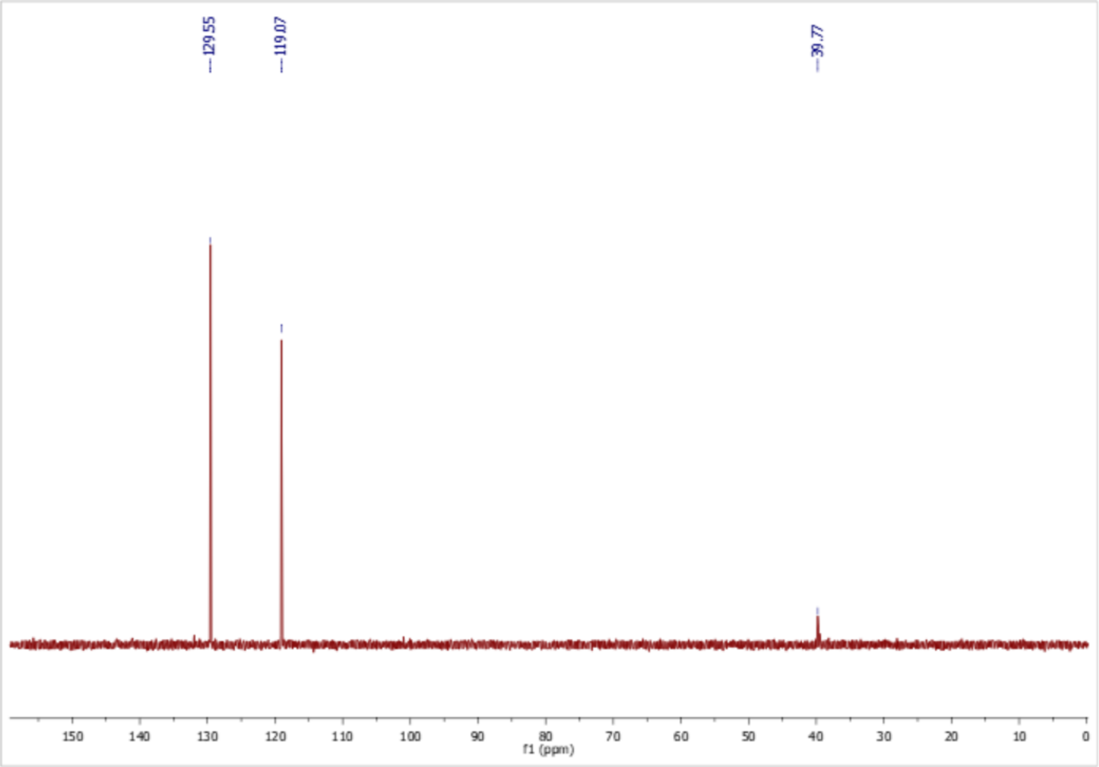

Figure S6. DEPT-135 of 7-bromo-4-chloro-1H-indazole-3-amine (**6**) in DMSO-d6.

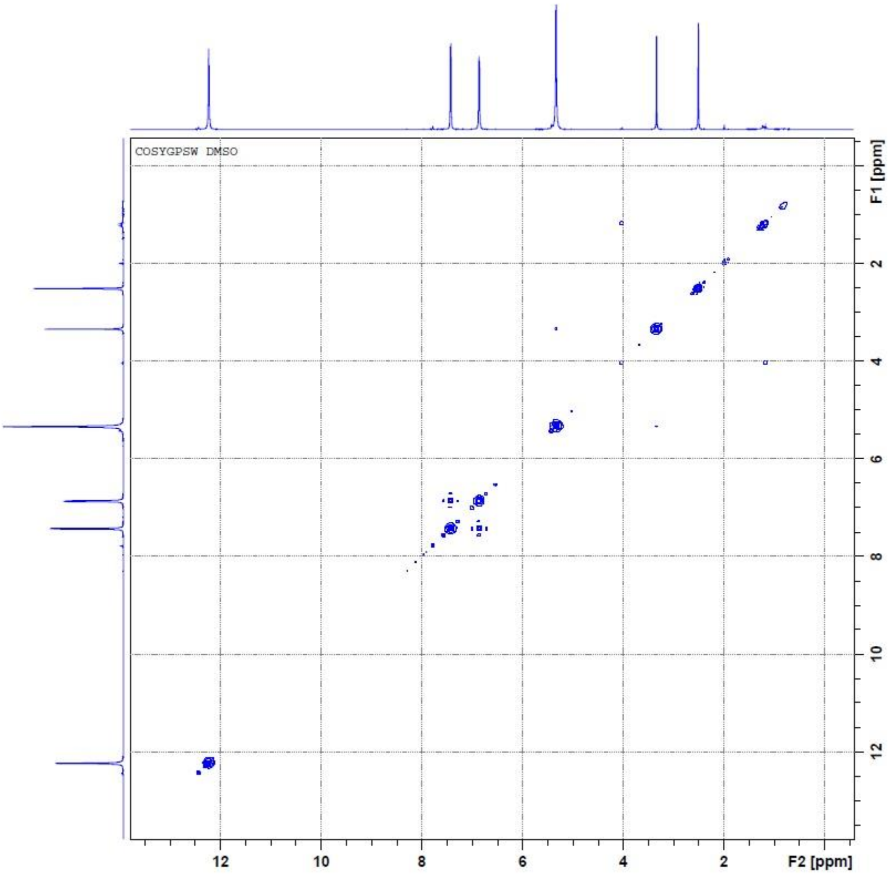

Figure S7. ^{1}H-^{1}H COSY of 7-bromo-4-chloro-1H-indazole-3-amine (**6**) in DMSO-d6.

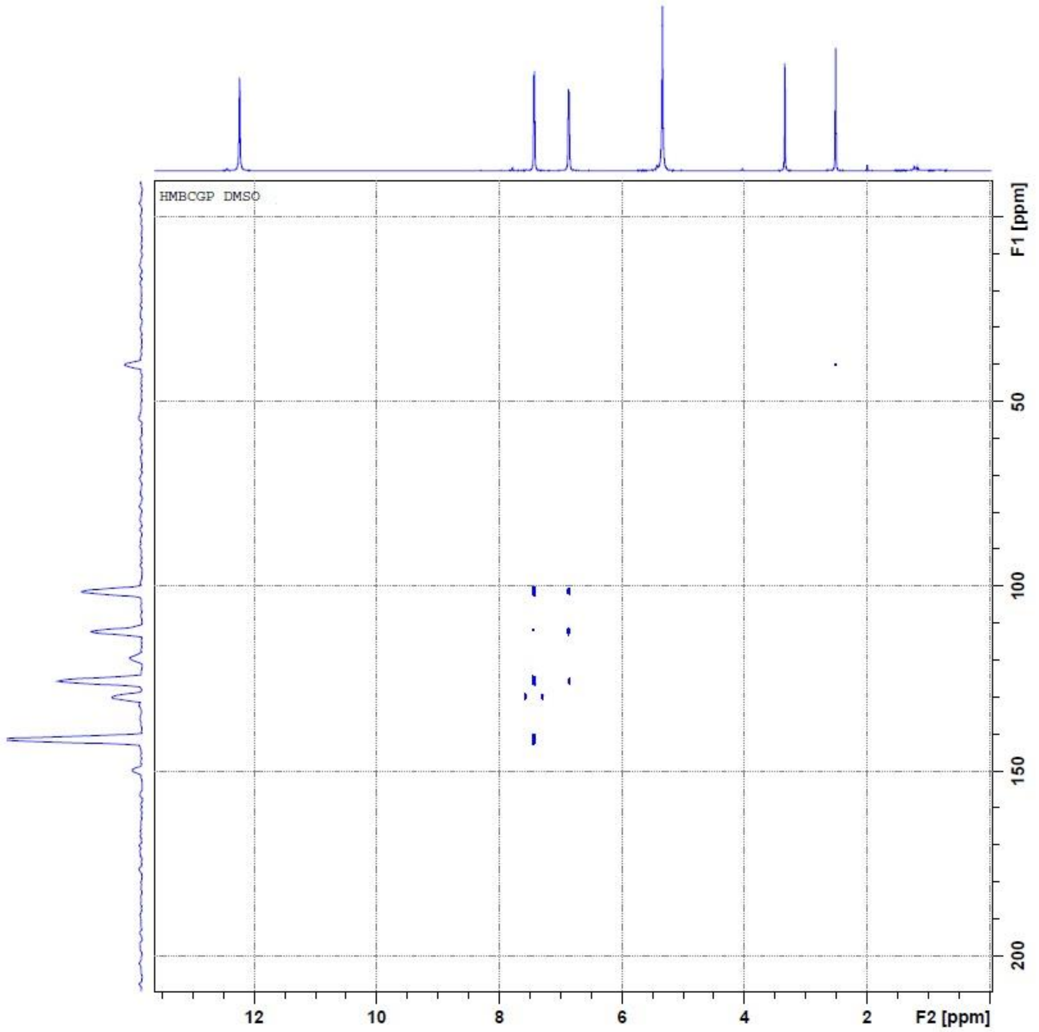

Figure S8. HMBC of 7-bromo-4-chloro-1H-indazole-3-amine (**6**) in DMSO-d6.

5-bromo-4-chloro-1H-indazol-3-amine (12)

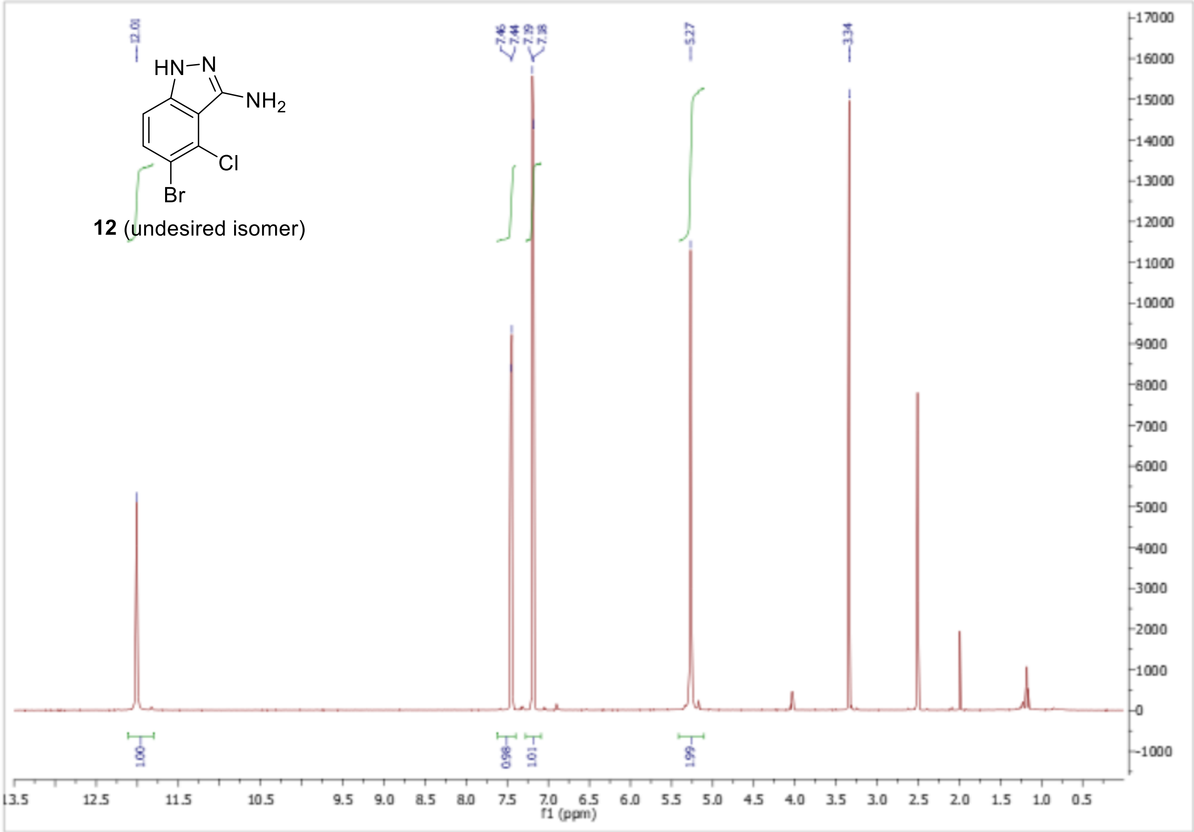

Figure S9. ^{1}HNMR of 5-bromo-4-chloro-1H-indazole-3-amine (**12**) in DMSO-d6.

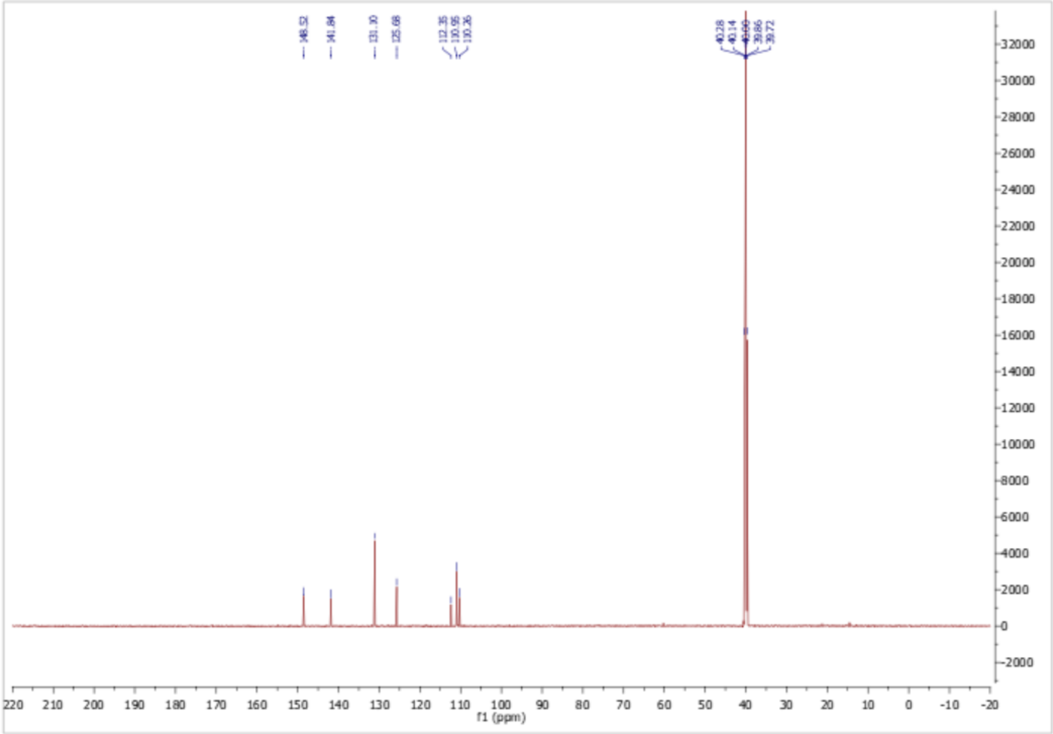

Figure S10. ^{13}CNMR of 5-bromo-4-chloro-1H-indazole-3-amine (**12**) in DMSO-d6.

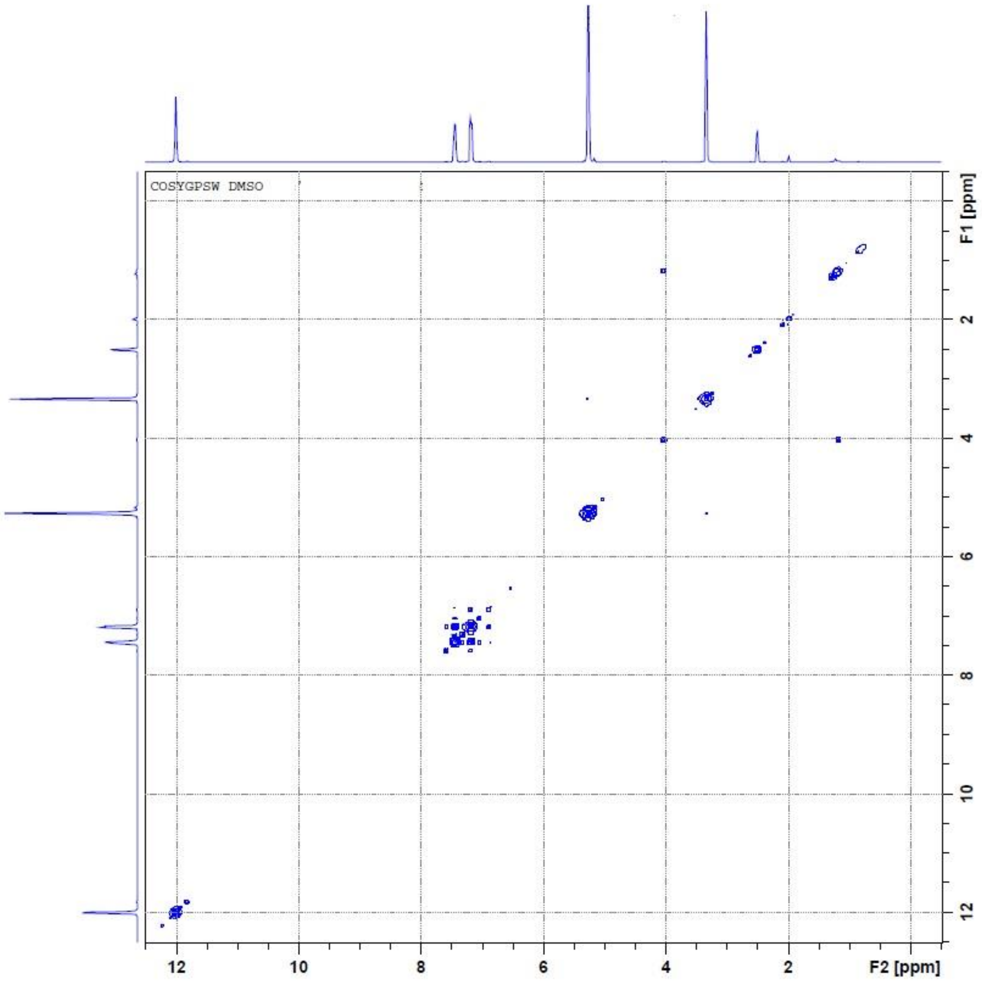

Figure S11. ^{1}H-^{1}H COSY of 5-bromo-4-chloro-1H-indazole-3-amine (**12**) in DMSO-d6.

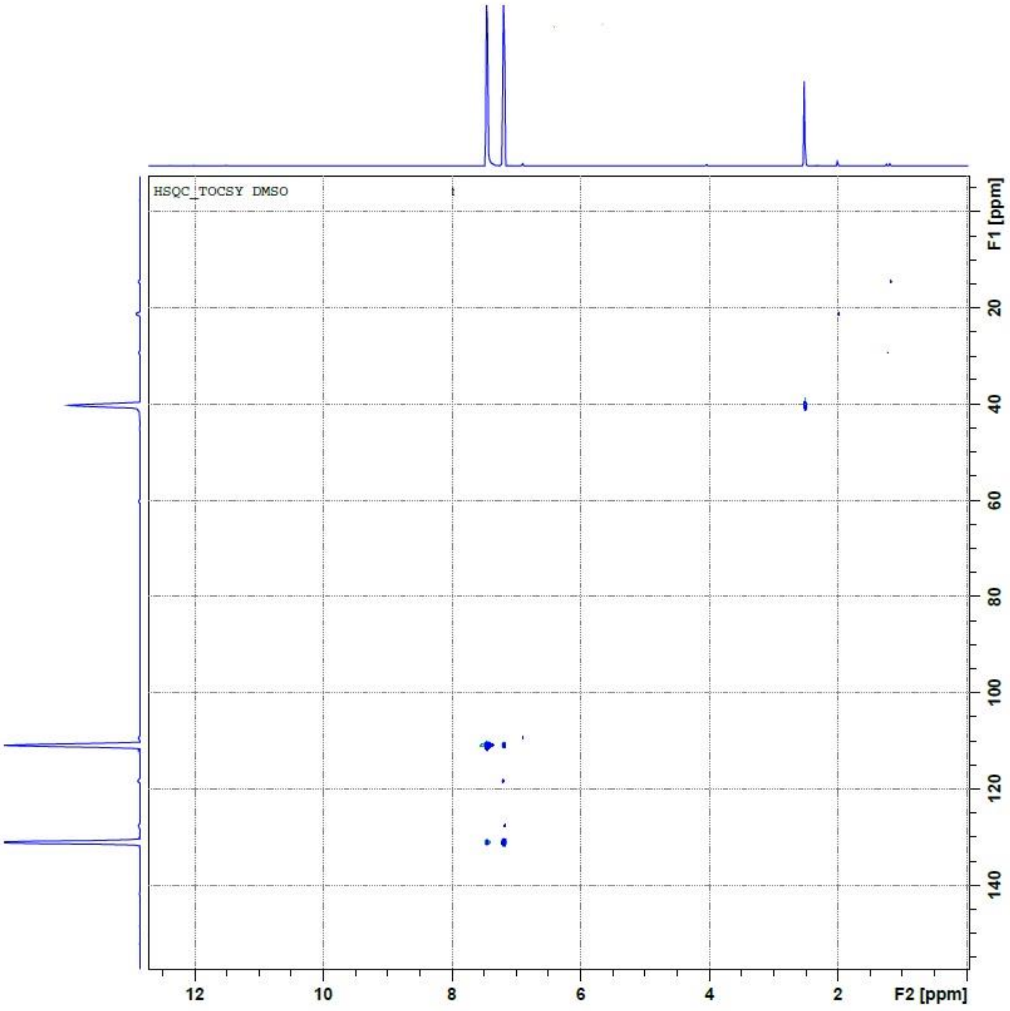

Figure S12. HSQC of 5-bromo-4-chloro-1H-indazole-3-amine (**12**) in DMSO-d6.

4-chloro-1H-indazol-3-amine (9)

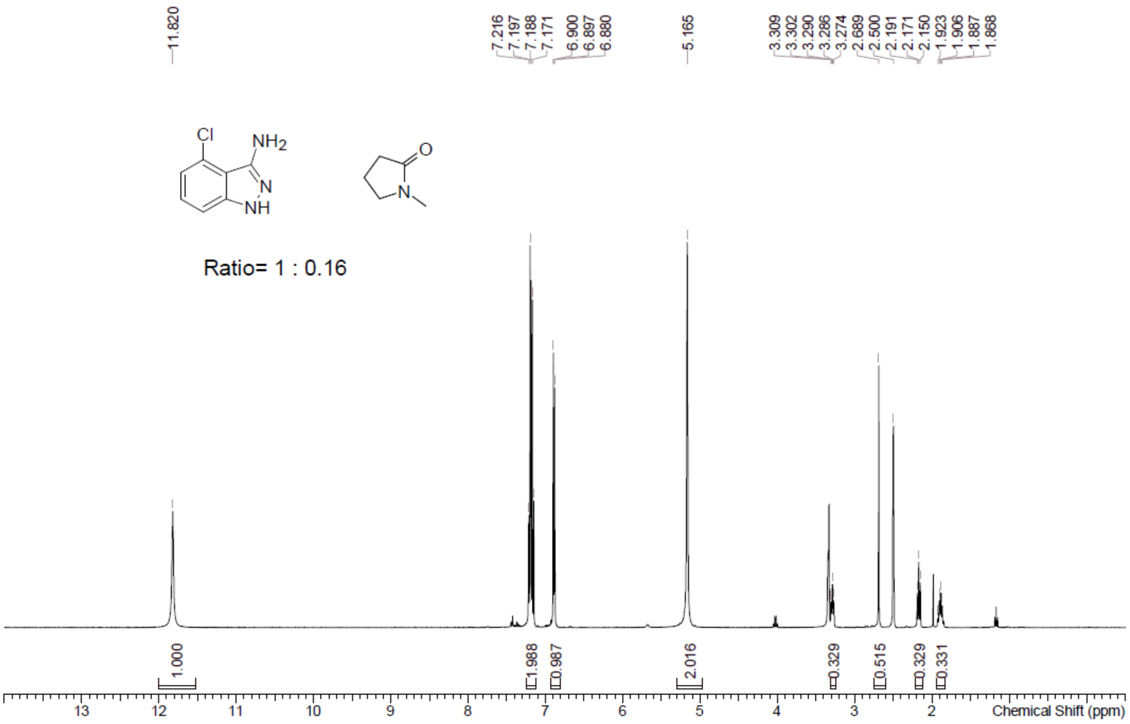

Figure S13. ^{1}HNMR of 4-chloro-1H-indazole-3-amine (**9**) in DMSO-d6 (400MHz).

1-(3-(bis(trimethylsilyl)amino)-4-chloro-1H-indazol-1-yl)-2,2-dimethylpropan-1-one (10a)

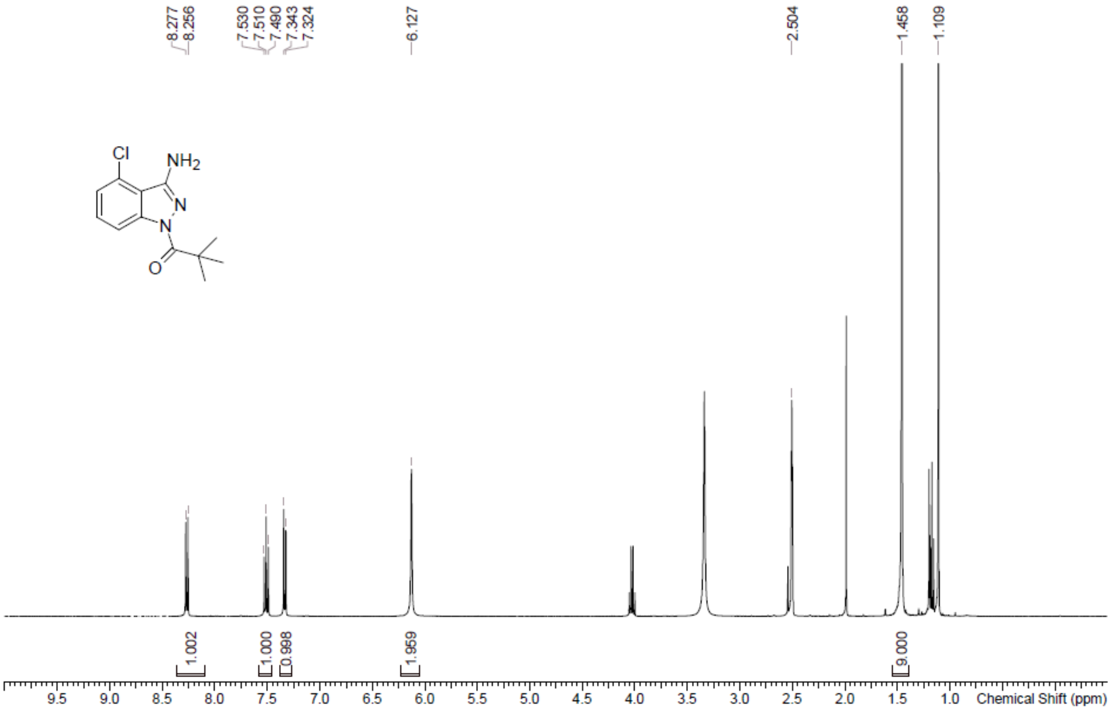

Figure S14. ^{1}HNMR of **10a** in DMSO-d6 (400MHz).

1-(3-(bis(trimethylsilyl)amino)-4-chloro-1H-indazol-1-yl)-2,2-dimethylpropan-1-one (10)

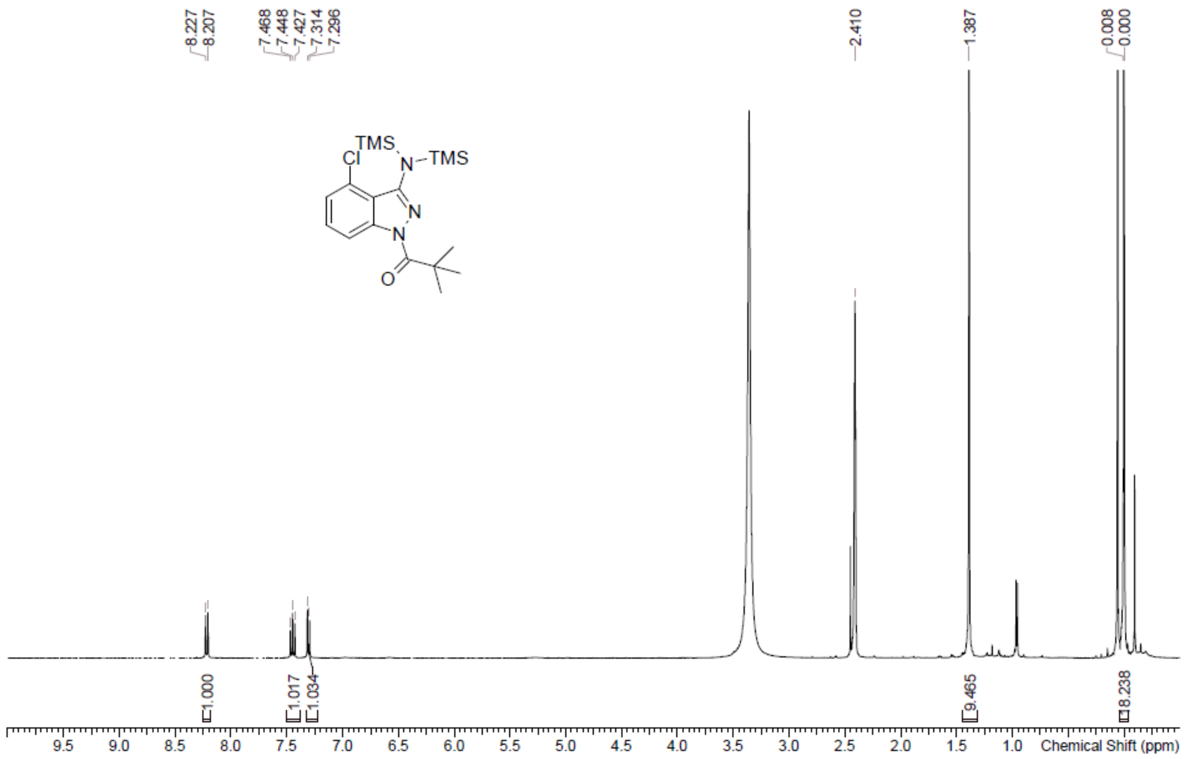

Figure S15. ^{1}HNMR of **10** in DMSO-d6 (400MHz).
